# Mast cell tolerance in the skin microenvironment to commensal bacteria is controlled by fibroblasts

**DOI:** 10.1016/j.celrep.2023.112453

**Published:** 2023-04-28

**Authors:** Anna Di Nardo, Yu-Ling Chang, Shahrzad Alimohammadi, Kana Masuda-Kuroki, Zhenping Wang, Krishna Sriram, Paul A. Insel

**Affiliations:** 1 Department of Dermatology, School of Medicine, University of California San Diego, La Jolla, CA 92037, USA; 2 Department of Pharmacology, School of Medicine, University of California San Diego, La Jolla, CA 92037, USA; 3 Department of Medicine, School of Medicine, University of California San Diego, La Jolla, CA 92037, USA; 4 Lead contact

## Abstract

Activation and degranulation of mast cells (MCs) is an essential aspect of innate and adaptive immunity. Skin MCs, the most exposed to the external environment, are at risk of quickly degranulating with potentially severe consequences. Here, we define how MCs assume a tolerant phenotype via crosstalk with dermal fibroblasts (dFBs) and how this phenotype reduces unnecessary inflammation when in contact with beneficial commensal bacteria. We explore the interaction of human MCs (HMCs) and dFBs in the human skin microenvironment and test how this interaction controls MC inflammatory response by inhibiting the nuclear factor κB (NF-κB) pathway. We show that the extracellular matrix hyaluronic acid, as the activator of the regulatory zinc finger (de)ubiquitinating enzyme A20/tumor necrosis factor α-induced protein 3 (TNFAIP3), is responsible for the reduced HMC response to commensal bacteria. The role of hyaluronic acid as an anti-inflammatory ligand on MCs opens new avenues for the potential treatment of inflammatory and allergic disorders.

## In brief

Di Nardo et al. find that mast cells in the skin tolerate skin commensal bacteria thanks to crosstalk with dermal fibroblasts that modulate mast cell reactivity by upregulating the A20 inhibitor of NF-κB. This mechanism maintains skin homeostasis while allowing mast cell response to other insults.

## Graphical Abstract

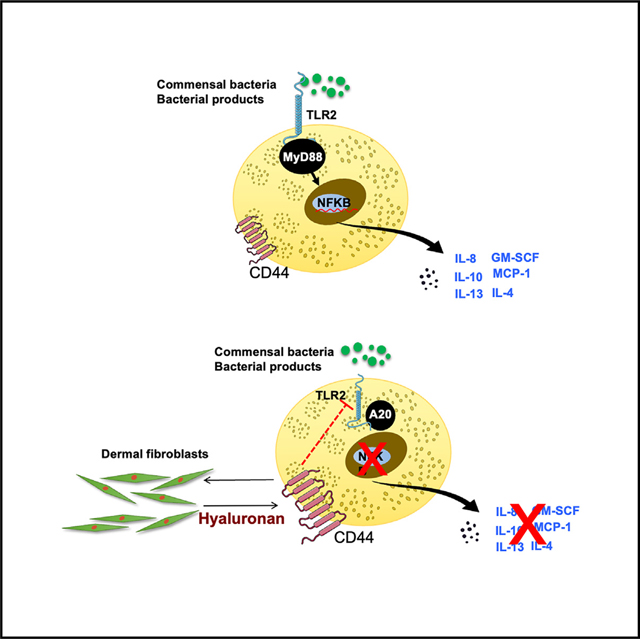

## INTRODUCTION

Mast cells (MCs) act as sentinels of the immune system^1,2^ at the body interface, especially in the gut and the skin.^3–10^ Immunoglobulin E (IgE) antibodies bind to MCs and basophils, the innate granulocytic effector cells of anaphylaxis. When the allergen- and cell-bound IgE antibodies interact, prostaglandin and leukotriene mediators, which are granular contents of these cells, are rapidly produced by the same cells and are released. They affect many tissue targets and trigger prompt physiologic responses and have been shown to be important mediators of innate and adaptive immunity.^11,12^

Abundant evidence shows that MCs can regulate immune responses through the production and release of cytokines and chemokines. MCs are activated by various stimuli, including microorganisms and their by-products. Many studies have investigated how MCs respond to bacteria *in vitro*. Experiments in MC-deficient mice have demonstrated the importance of MCs in protecting against skin infections,^8,9,13^ but few studies have compared the behavior of MCs with bacteria *in vitro* with that *in vivo*.

MCs tolerate commensal bacteria and many other stimuli on the surface and are positively influenced by the skin microbiome.^14^ MC tolerance to the external environment is key to skin homeostasis, but the mechanism that allows this tolerance has not been defined.

Fibroblasts are known to have different genetic signatures depending on the tissues in which they reside. It has been suggested that MC communication with stromal cells or fibroblasts induces optimal, tissue-dependent maturation. Indeed, co-culture of skin 3T3 fibroblasts with immature MCs modulates the MC phenotype by inducing expression of proteases and adhesion molecules.^15^ In addition, morphological and biochemical analyses reveal that skin fibroblasts regulate the expression of secretory granule components, such as heparin and chondroitin sulfate, in co-cultured MCs. Thus, co-culture with stromal cells or fibroblasts adjusts the MC receptor expression profile based on the local cellular environment, resulting in the terminal differentiation of MCs.^16^

In this study, we tested the hypothesis that (1) the immunological mechanism responsible for dampening human MC (HMC) activation at the skin interface is due to the interactions of MCs with dermal fibroblasts (dFBs) and (2) this dampening is part of a comprehensive anti-inflammatory activity that dFBs exert on HMCs in the dermis. We describe here how our initial finding that HMCs conditioned by dFBs become tolerant to commensal bacteria led us to discover a broader system through which dFBs control multiple MC pathways and receptors to prevent unnecessary inflammation. By employing bulk and single-cell RNA sequencing (RNA-seq) analysis, we identified unique genes that dFBs express to reduce pro-inflammatory cytokine release from HMCs.

In addition, we discover that the production by dFBs of hyaluronan (HA), via Toll-like receptor 2 (TLR2) downregulation, is critical for regulating innate immunity in the skin. MCs express multiple pattern recognition receptors (PRRs), including TLRs on their cell surface, to detect harmful antigens that come in contact with the host.^10,17–19^ TLR2 and TLR4 are particularly important signaling receptors for multiple microbial components and are considered essential for mediating host immune response against major infectious diseases.^20–22^

We thus discovered a critical, previously unrecognized mechanism by which dFBs modulate response to commensal bacteria in MCs: production by dFBs of HA activates A20/tumor necrosis factor α-induced protein 3 (TNFAIP3) and increases nuclear factor κB (NF-κB) inhibitors in the cytoplasm that limit NF-κB gene expression, thereby modulating the release of inflammatory cytokines on MCs. TLR2 and CD44 communication is another mechanism that contributes to blunting of TLR activation by commensal bacteria. The current discovery reveals that dFB-MC interaction in the dermis makes MCs tolerant to commensal bacteria and immunotolerant to the skin environment.

## RESULTS

### HMC interactions with dFBs modulate MC reactivity to bacteria

**Figure 1.**
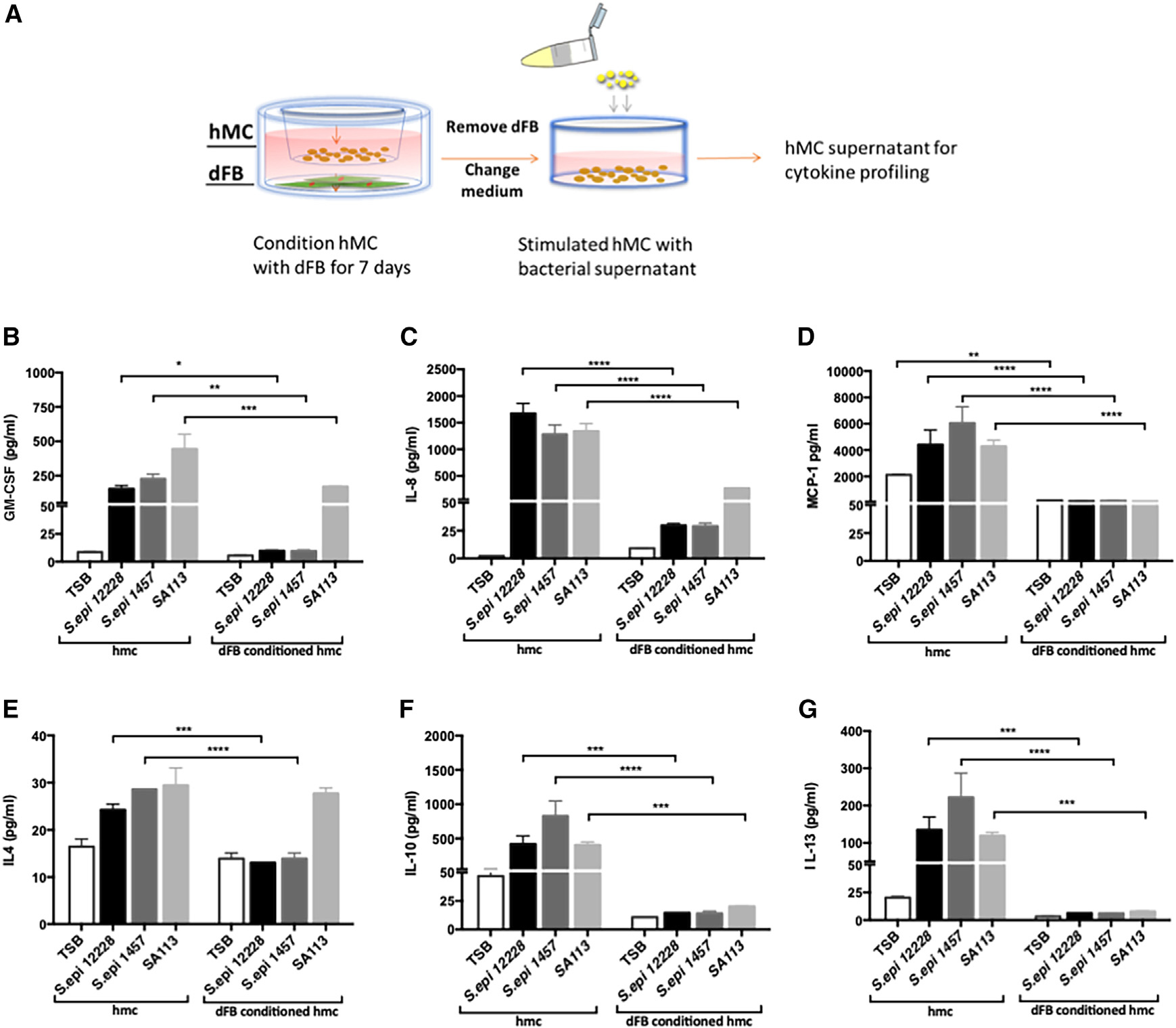
Human mast cell (HMC) interactions with dermal fibroblasts (dFBs) modulate MC reactivity to bacteria (A) Human MCs were conditioned by co-culturing with human dFBs for 7 days using cell culture inserts. Unconditioned HMCs or dFB-conditioned HMCs were stimulated with TSB, a filtered bacteria supernatant of *S*. *epi* 12228, *S*. *epi* 1457, or SA113 (*S*. *aureus)* for 24 h. (B–G) Protein quantification of pro-inflammatory cytokines released including GM-CSF, IL-8, and MCP-1 (B–D) and anti-inflammatory cytokines IL-4, IL-10, and IL-13 (E–G) were measured by Luminex multiplex. Statistical significance of mean ± SD was calculated via unpaired ANOVA. *p < 0.05; **p < 0.01; ***p < 0.001 (n = 3). (B)–(G) are representatives of biological replicates.

To determine whether MC interaction with dFBs contributes to the suppression of MC reactivity to commensal bacteria, we cultured HMCs in the presence or absence of dFBs. Seven days after co-culture, we challenged unconditioned and dFB-conditioned HMCs with supernatant from two commensal bacteria, *S*. *epidermidis* 12228 *(S. epi* 12228) and *S*. *epidermidis* 1457 (*S*. *epi* 1457) and, from an opportunistic strain, *S*. *aureus* (SA113). Following this challenge with a bacterial supernatant, we used an ELISA (Luminex multiplex assay) to evaluate secreted pro- and anti-inflammatory cytokines (Figures 1 and S1). The cytokine profile revealed that both commensal and opportunistic bacterial supernatantactivated unconditioned HMCs and that certain cytokines released by unconditioned HMCs were downregulated in dFB-conditioned HMCs. The downregulated cytokines included the pro-inflammatory cytokines GM-CSF, interleukin-8 (IL-8), IL-4, and MCP-1 (Figures 1B–1D) and the anti-inflammatory cytokines IL-10 and IL-13 (Figures 1E–1G). In addition, dFB-conditioned HMCs had a reduced response to bacterial supernatant that was greater for commensal than opportunistic bacteria *(S. aureus)* supernatant. These findings led us to hypothesize that crosstalk from dFBs dampened the MC response to commensal bacteria and modulated the response to more dangerous bacteria.

### The skin environment reduces MC expression of TLR2 receptors

**Figure 2.**
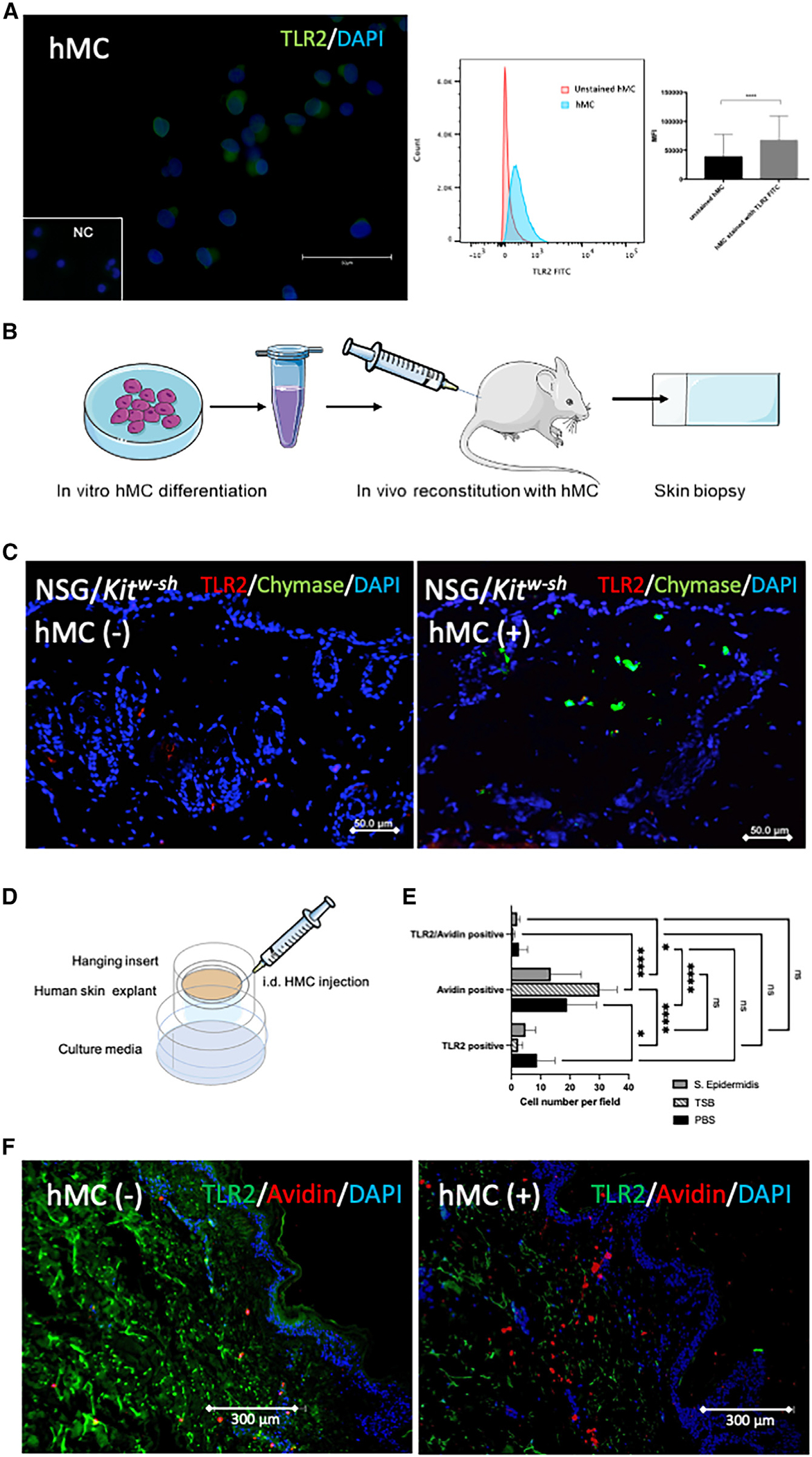
dFBs are responsible for reducing MC expression of TLR2 receptors (A) Immunofluorescence staining of FITC-conjugated TLR2 (green), and DAPI (blue) on HMCs (left), FACS histogram of unstained and FITC-conjugated TLR2 stained HMCs (middle), and FACS MFI (triplicates) for TLR2 (right). (B) Diagram of *In vitro* reconstitution of HMC into mouse skin. Scale bar: 50 μm. (C) TLR2/chymase immunofluorescent staining of mouse skin reconstituted with HMCs; control staining, NSG^−/−^ (NOD SCID gamma immunedeficiency mouse), Kitw-sh^−/−^ (MC-deficient mouse), and HMC intradermal reconstitution in NSG/Kitw-sh. Green: chymase; red: TLR2. Scale bar: 50 μm. (D) Diagram of HMC-injected and *S. epi*-treated human skin explants (Genoskin). (E) Quantification of HMCs (avidin positive), of HMCs expressing TLR2 (TLR2 and avidin positive), and cells expressing only TLR2 (not HMCs) expressed as number of cells for microscope field at 10×. Red: avidin; green: TLR2. Statistical significance was calculated via unpaired ANOVA. *p < 0.05; **p < 0.01; ***p < 0.001. (F) TLR2/avidin immunofluorescent staining in human *ex vivo* skin (Genoskin) injected with mature HMCs. *Ex vivo* skin section after application of *S. epi* and HMC injection to upper skin and dermis. Figures show biological triplicates. Scale bar: 300 μm.

As the frontline of host defense in the skin, HMCs express PRRs (e.g., TLR2), which trigger inflammatory mediators involved in eliminating invasive threats.^23–26^ Since TLR2 is a receptor triggered by both skin commensal and pathogenic bacteria, it is essential to evaluate TLR2 expression in MCs in the dermal environment. Previous studies have shown that mouse MCs highly express TLR2 receptors *in vitro* but rapidly lose them in the skin microenvironment.^14,27^ However, little is known about HMCs and their TLR expression in the skin environment. To confirm TLR2 expression on HMCs, we investigated its presence by flow cytometry analysis (fluorescence-activated cell sorting [FACS]) and immunofluorescence (IF) imaging. HMCs were stained with fluorescein isothiocyanate (FITC)-conjugated antibodies; Figure 2A represents IF imaging (left), FACS histogram (middle), and mean fluorescence intensity (MFI) triplicates of FACS analysis (right).

To confirm the *in vivo* TLR2 expression changes on HMCs in the dermis, we used NOD SCID gamma (NSG) mice, an immunodeficient mouse strain that can be used as a humanized mouse for *in vivo* study. NSG mice are deficient in mature T cells, B cells, and natural killer (NK) cells and signaling pathways for multiple cytokines. However, they have mouse MCs. Therefore, we bred NSG mice with c-Kit^*W-sh*^ mice, resulting in immunodeficient and MC-deficient mice (NSG/c-Kit^*W-sh*^). This produced a functional humanized immune system for HMCs in NSG mice. After we confirmed TLR2 expression in cord blood-derived HMCs *in vitro* (FACS data, Figure 2A), we injected NSG/c-Kit^*W-sh*^ mouse skin intradermally with HMCs, waited 15 days for reconstitution of the HMC population, and then assessed murine skin sections by IF. We observed that TLR2 was not co-localized with the chymase signal from HMCs in the skin (Figure 2C). HMCs express TLR2 *in vitro,* but TLR2 expression disappeared *in vivo.*

To further confirm that TLR2 expression changes on HMCs *in vivo* in the dermis, we injected 1 × 10^6^ HMCs into human skin samples (*ex vivo* preparation from Genoskin) and incubated the explant for 4 days. Genoskin allows for assaying live skin biopsies that exhibit normal skin barrier function and contain all cell types naturally present in *in vivo* human skin (Figures 2D–2F). The explants did not show a significant number of MCs at baseline or after injection with PBS. After 4 days of incubation, we processed the skin for paraffin embedding and immunofluorescent staining for TLR2 and c-kit, an MC marker. As we found in the mouse reconstitution experiment, MCs reduced the expression of TLR2 receptors in the dermal environment (Figure 2F). These data thus confirm that HMCs downregulate TLR2 expression in the dermal environment, both *in vitro* and *in vivo.*

### Dermal MC TLR2 expression is mirrored by the cytokine response to lipoteichoic acid (LTA)

**Figure 3.**
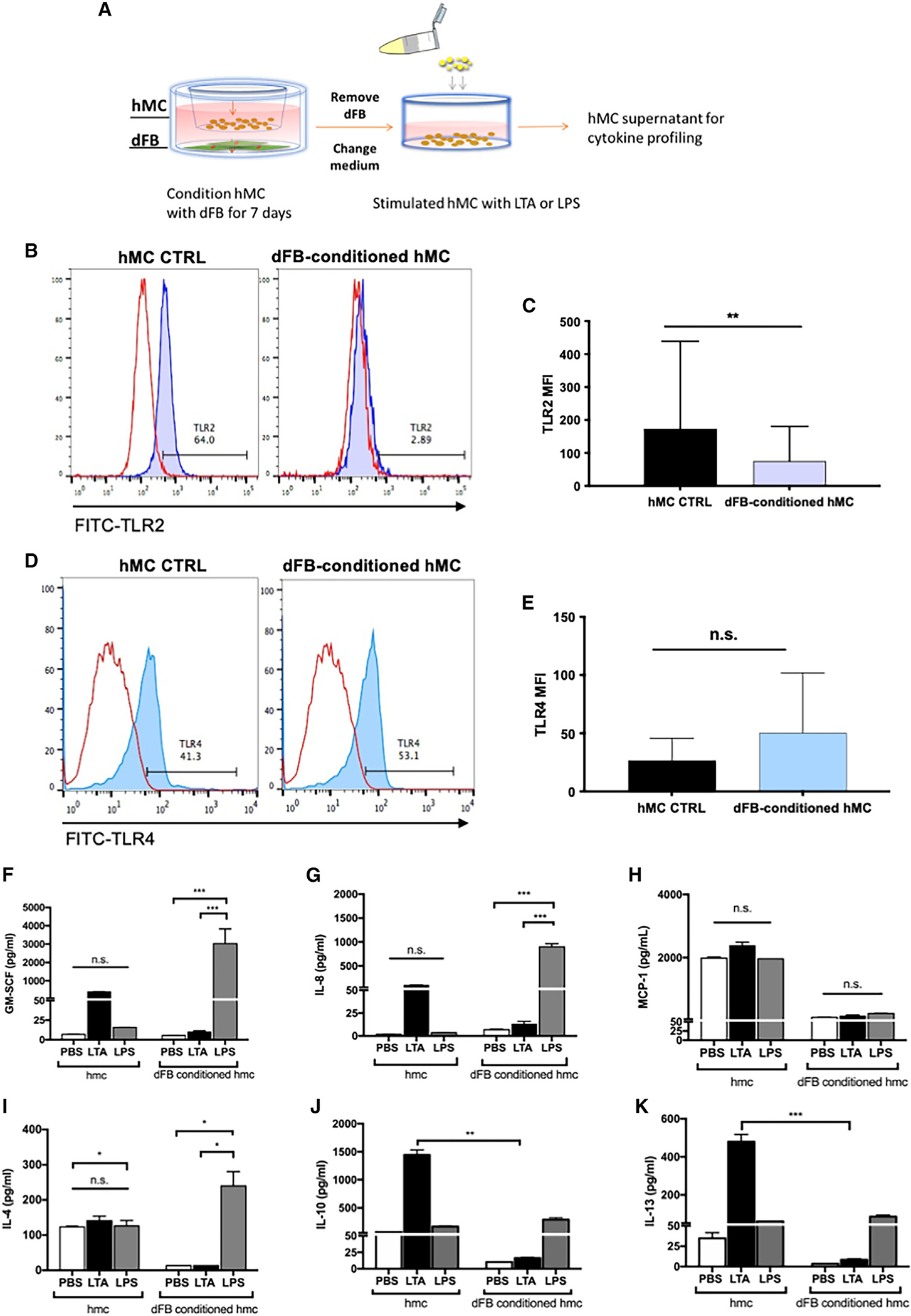
Dermal MC TLR2 expression is mirrored by LTA cytokine response (A) Flow cytometry analysis of diagram of HMCs conditioned by co-culturing with dFBs for 7 days using cell culture inserts. The medium and dFBs were removed, and HMC supernatants were used for cytokine profiling. (B) TLR2 expression in HMCs after dFB-conditioning by FACS. (C) TLR2 MFI of HMCs and dFB-conditioned HMCs. The data shown represent the mean ± SD (n = 3). Statistical significance was calculated via paired t test; **p < 0.01. (D) TLR4 expression in HMCs after dFB conditioning by FACS. (E) TLR4 MFI of HMCs and dFB-conditioned HMCs. The data shown represent the mean ± SD (n = 3). Statistical significance was calculated via paired t test; n.s., not significant, figures show biological triplicates. (F–K) HMCs or dFB-conditioned HMCs were stimulated with PBS, LTA (10 μg/mL), or LPS (100 ng/mL) for 24 h. Protein quantification of pro-inflammatory cytokines released including (F) GM-SCF. Statistical significance was calculated via unpaired ANOVA. *p < 0.05; **p < 0.01; ***p < 0.001. (G) IL-8, (H) MCP-1, and anti-inflammatory cytokines (I) IL-4, (J) IL-10, and (K) IL-13 were measured by Luminex multiplex. In (F)–(K), statistical significance was calculated via unpaired ANOVA. *p < 0.05; **p < 0.01; ***p < 0.001. (F)–(K) are representatives of biological replicates.

To further examine how the dFB culture environment affects the expression of TLRs by MCs, we co-cultured MCs with dFBs, and following co-culture, we stimulated HMCs with TLR2 and TLR4 ligands, LTA, and lipopolysaccharide (LPS), respectively, as described in the diagram of Figures 3A. After dFB conditioning, HMC expression of TLR2 decreased from 64% to 2.9% (Figures 3B and 3C), while TLR4 expression increased from 41% to 53% in dFB-conditioned HMCs compared to HMCs alone (Figures 3B–3E and S8).

We then characterized the cytokines generated by MCs from unconditioned and dFB-conditioned HMCs. We observed that unconditioned HMCs are very responsive to LTA stimulation (Figures 3F–3K and S1) and that GM-SCF, IL-8, MCP-1, IL-10, and IL-13 showed noticeable changes in expression (Figures 3F–3K). However, after dFB conditioning, those cytokines were downregulated in response to LTA stimulation. This trend was not observed if HMCs were stimulated with LPS, a key component of Gram-negative pathogenic bacteria, such as *E. coli* and *Pseudomonas,* which are not commonly resident in the skin. Stimulation with LPS of dFB-conditioned HMCs increased the expression of cytokines, including GM-SCF, IL-8, and IL-4. These cytokines play important roles in recruiting other immune cells, including macrophages, neutrophils, and Th2 cells. Notably, the TLR4 receptors on HMCs slightly changed after dFB conditioning (Figures 3D, 3E, and S8). Thus, interaction with dFBs modulates HMCs *in vitro* and elicits different responses to different TLRs: tolerance to the presence of Gram-positive bacterial cell components recognized by TLR2 while maintaining the inflammatory response to specific pathogens that TLR4 recognizes. We investigated if TLR2 ligands other than LTA (SE12228, Pam2CSK4, and Zymosan) showed similar effects on HMCs. As assessed by IF, adding these ligands decreased TLR2 expression (Figure S9). We next investigated possible mechanisms underlying dFB crosstalk with MCs based on these findings.

### scRNA-seq showed that MC and dFB gene expression profiles are significantly changed by co-culture

**Figure 4.**
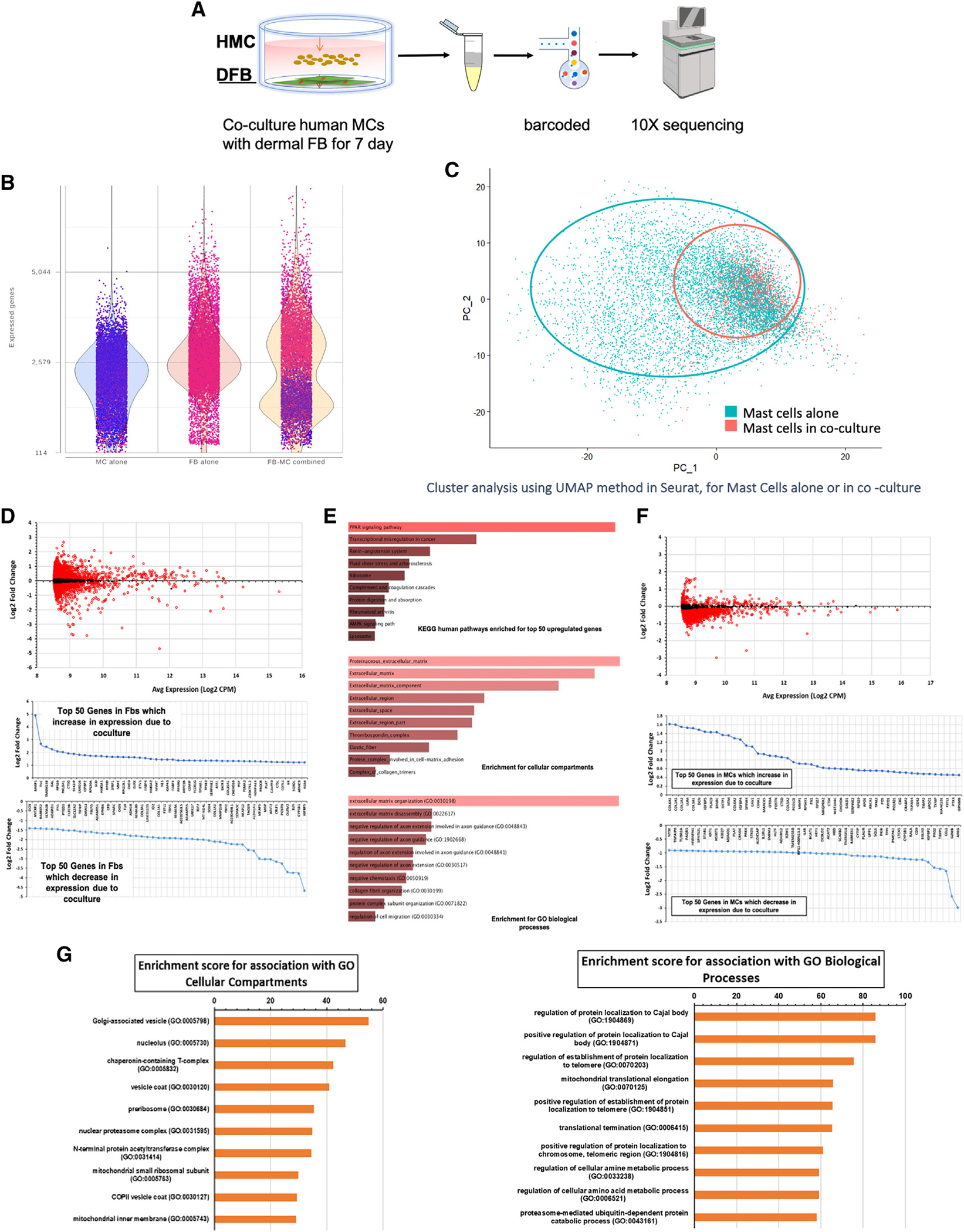
Both MC and dFB genetic profiles are significantly changed when co-cultured (A) Diagram of single-cell RNA-seq analysis of changes of HMCs and dFBs cultured alone or in co-culture. (B) Quantity of genes expressed in HMC and dFB populations before and after co-culture. (C) Cluster analysis of HMCs alone (blue) and HMCs co-cultured with dFBs (orange). Both cell populations represented in UMAP1 and UMAP2 show a shift that is more pronounced for the dFB population. (D) Most significant genes in HMC and dFB populations changed by co-culture. Top panel shows the DE of dFBs in co-culture vs. alone. DE analysis results for dFBs showed 7,802 genes with a significant change (FDR < 0.01), with 95 significantly increased >2-fold in co-culture, 104 significantly decreased >2-fold in coculture, 1,086 significantly increased >50% in co-culture, and 464 significantly decreased >50% in co-culture. A positive value change shows higher expression in co-culture (FDR < 0.01). (E) Pathway analysis for dFB-upregulated genes: downregulated genes in dFBs appear to be associated with ECM and fibrotic markers, and the dFBs are less “fibrotic” in co-culture. Upregulated genes in dFB appear to be related to PPAR, most likely PPARγ, which has increased expression. (F) DE analysis of increased and decreased genes in HMCs by co-culture. DE analysis results for HMCs showed the number of significant genes (FDR < 0.01) was 8,840 with 15 significantly increased >2-fold in co-culture, 25 significantly decreased >2-fold in co-culture, 59 significantly increased >50% in co-culture, and 2,172 significantly decreased >50% in co-culture. (G) HMC enrichment score for cellular compartment and biological process relative to the HMC genes changed by co-culture.

To understand how dFB modulates MC response to bacteria and induces selective tolerance to commensal bacteria, we compared the gene expression profiles in both cell types under different conditions (Figure 4B). We first subjected unconditioned and dFB-conditioned MCs to single-cell RNA-seq (scRNA-seq) analysis and evaluated the genes’ differential expression (DE) in each case. Using Fisher’s exact test, we compared both groups by detected and undetected contingency tables. This non-parametric test is appropriate, as it carries the fewest assumptions regarding the underlying statistical behavior of the data, assumptions that make it inappropriate to use specific other statistical analyses, such as t tests. This approach yields odds ratios that indicate whether a gene is more likely to be expressed in one group or the other and gives a corresponding p value for each gene. Values were adjusted for multiple testing in R programming, using false discovery rates (FDRs); FDRs <0.01 were considered significant. Therefore, we tested for the presence/absence of genes between groups, rather than differences in quantity, due to the essentially binary nature of the output data (details are in Figure S2).

Cluster analysis of genetic profiles indicated that dFBs co-cultured with MCs changed dramatically; in parallel, the profile of MCs shifted (Figure 4C). The two cell populations thus strongly influenced each other. We applied the selector tool in Seurat and marked the subpopulations for further DE analysis (Figures 4D and S2).

#### Fibroblast changes

DE analysis of dFBs identified 7,802 genes with a significant change (FDR < 0.01), with 95 genes having a >2-fold increase and 104 genes with a >2-fold decrease in expression in response to co-culture. Genes whose expression was increased or decreased >50% with co-culture numbered 1,086 and 464, respectively. Downregulated genes in dFBs are associated with extracellular matrix (ECM) and fibrotic markers; the dFBs are less “fibrotic” in co-culture (Figure S5). Upregulated genes in dFBs appear to be related to PPAR signaling, most likely PPARγ, which is itself upregulated in expression, along with matrix metalloproteinase 1 (MMP-1) and IL-1R1 (Figures 4D and 4F).

#### HMC changes

In total, 8,869 genes had DE between HMCs grown alone or in dFB co-culture (FDR < 0.01). Genes whose expression was increased or decreased >50% with co-culture numbered 59 and 2,172, respectively, indicating that co-culture induced an overall loss in the repertoire of genes expressed in HMCs. The downregulated genes are associated with a range of immune and inflammatory signaling pathways, including IL-2 and IL-6, in addition to mTORC1 and NF-κB signaling (Figure 4G). By contrast, the genes that increased in expression are associated with the ECM, including numerous collagen genes. Thus, HMCs show reduced expression of genes/pathways typically associated with immune activation and function of MCs and other immune cell types (Figure S5).

### Bulk RNA-seq analysis showed that dFB-conditioned HMCs upregulate genes that inhibit the NF-κB pathway

**Figure 5.**
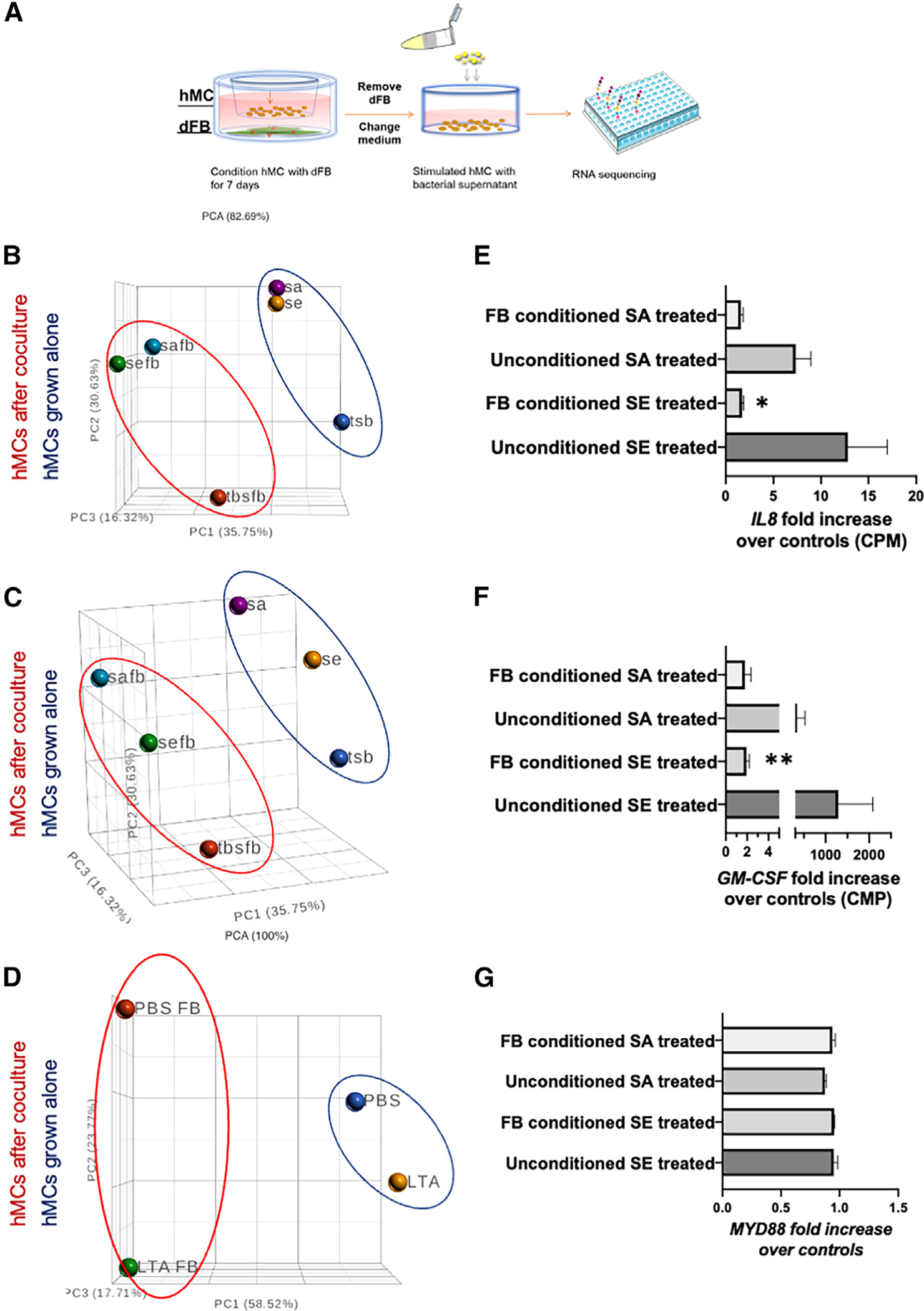
Principal-component analysis cluster plotters of HMCs conditioned with dFBs or not conditioned (A–D) Diagram of the experiment relative to the RNA-seq of HMCs conditioned with dFBs or not conditioned for 7 days (A), treated with bacterial supernatant (B and C) or LTA as TLR2 ligand (D). (E–G) RNA-seq data expressed in CPM; data are triplicate values and expressed as fold increase vs. control cells not stimulated with the bacterial supernatant *S*. *epi* (SE) or *S*. *aureus* (SA). *p < 0.05 using gene-specific analysis (GSA) in Partek software. Triplicate data.

We sought to determine the pathways most associated with the significantly changed genes expressed in dFB-MC co-culture in response to bacterial exposure. We performed bulk RNA-seq analysis of MCs stimulated with Gram-positive bacterial supernatant in the presence and absence of dFBs. We co-cultured MCs for a week with dFBs, separated them, and challenged them with LTA, *S*. *epi,* and SA113. The controls were PBS for LTA and tryptic soy broth (TSB) for bacteria supernatants (Figure 5B). Principal-component analysis (PCA) of the samples indicated that dFB conditioning was the main factor in changing the mRNA profile of the MCs (Figures 5B–5D, S3, and S4). Adding the bacterial supernatant further changed the mRNA cell profile (3D PCA, Figures 5B and 5C) compared with control MCs and MCs conditioned with dFBs. The PCA of cells treated with LTA differed from that of the cells treated with bacterial supernatant (3D PCA, Figure 5D).

From the same RNA-seq data, we analyzed the fold increase of mRNA from supernatant-treated cells compared with non-treated cells associated with the cytokines that were identified by ELISA Luminex analysis (Figures 5E–5G). We found that IL-8 decreased 8-fold (Figure 5E), and GM-CSF decreased >10-fold (based on DE analysis of bulk RNA-seq data, Figures 5F) in the conditioned, treated cells. By contrast, expression of MYD88, a cytosolic adapter protein that is activated by TLR2, was unchanged by dFB conditioning or if we included *S*. *epi* or SA113 in the cultures (Figure 5G). Additional statistical volcano graphs are in supplemental information (Figure S3).

**Figure 6.**
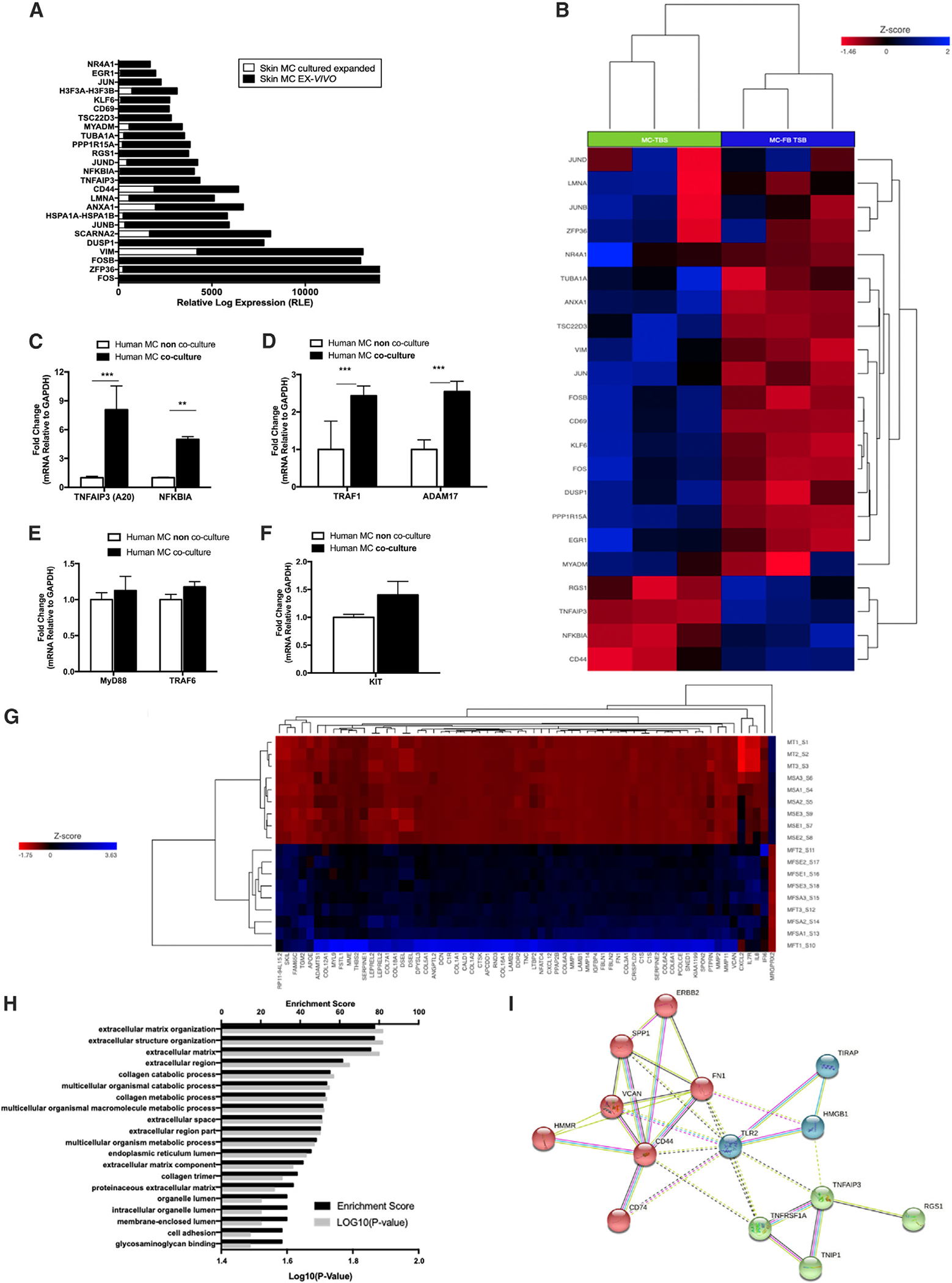
dFB-conditioned HMCs upregulate genes that inhibit the NF-κB pathway (A) Identification of unique genes exclusively modulated in the dermal environment from the FANTOM 5 data set. (B) Most differentially expressed genes from MC in skin *ex vivo.* Heatmap of the same genes in (A) from the RNA-seq of HMCs conditioned and not conditioned with dFBs for 7 days. (C–E) qPCR analysis of NF-κB, TNFAIP3 (A20), NFKBIA, TRAF1, ADAM17, MyD88, and TRAF6 expression from HMCs conditioned or not conditioned with dFBs *in vitro.* Statistical significance was calculated via unpaired ANOVA. *p < 0.05; **p < 0.01; ***p < 0.001 (n = 3, triplicates). (F) qPCR of c-kit gene in HMCs conditioned or not conditioned with dFBs. Changes are not significant. Statistical significance was calculated via unpaired ANOVA (n = 3, triplicates). (G) RNA-seq of HMCs conditioned or not conditioned with dFBs before treatment with TSB (MT and MFT), *S. epi* 12228 (MSE and MFSE), and *S. aureus* 131 (MSA and MFSA). The plot shows a significant decrease in MRGPRX2 in cells conditioned with dFBs. (H) Enrichment score of the most significant pathways affected by dFB conditioning (PathwayCommons.org). (I) K-mean cluster of the genes that are significantly increased after dFB conditioning and their correlation with TLR2 (String pathway).

To validate our *in vitro* system, we compared it with the *ex-vivo* FANTOM 5 consortium dataset, a standard for analyzing the transcriptome of quiescent HMCs in the dermis.^28^ The aim was to compare our dFB co-cultured HMCs with *ex vivo* dermal HMCs. We reanalyzed the FANTOM 5 database focusing on innate immune response genes and created a list of the 22 most expressed genes *in vivo* compared with *ex vivo.* We found that multiple genes that are highly expressed in *ex vivo* human skin MCs are associated with the NF-κB and MAPK pathways (Figure 6A). For instance, the FANTOM 5 transcriptome reveals that skin MCs *ex vivo* highly expresses TNFAIP3 (A20) and NFKBIA, but their expression gradually decreased in the FANTOM 5 expanded culture of human skin MCs (Figure 6A). TNFAIP3 (A20) and NFKBIA regulate NF-kB activation, downregulating TLR signals, and cytokine production.^29–31^

Analysis of RNA-seq data of HMCs before and after dFB co-culture for the same 22 genes with DE in the FANTOM5 set revealed that most of the modulated genes were similarly altered in our mRNA-seq analysis (Figure 6B). Few genes showed strong DE and included TNFAIP3, NFKBIA, and CD44, all inhibitory genes, as predicted by our prior scRNA-seq analysis, as shown above. PCR analysis confirmed that inhibitory genes associated with NF-κB inhibitory pathways were upregulated (Figures 6C and 6D) but that genes such as MyD88, TRAF6, and KIT, were not changed (Figures 6E and 6F). Of note, two other genes, RGS1 and CD44, were also significantly expressed in HMCs co-cultured with dFBs (Figure 6B). Flow cytometry measurements revealed that CD44 expression is decreased in dFB-conditioned HMCs compared with HMCs alone (Figure S6).

We compared our bulk RNA-seq data from unconditioned and dFB-conditioned MCs, selecting a 4-fold increase significance (p < 0.05) (Figure 6G). This analysis confirmed the previous data and additionally showed that MRGPRX2, a G protein-coupled receptor (GPCR) in MCs, was strongly negatively modulated by co-culture with dFBs (Figure 6G).

Inputting a list of significant genes into the pathwaycommons.org online tool for analysis (Figure 6H) revealed that the great majority of the genes with the highest expression scores were those in the ECM, a result confirmed by the “string pathway” software tool (Figure 6I). We found that the genes clustered within three pathways (CD44, TLRs, and A20/TNFAIP3) with TLR2 at the center (Figure 6I). Based on the results from the single cells and the bulk RNA-seq, we decided to investigate ECM components further.

### HA, an extracellular matrix component, inhibits MC cytokine release

**Figure 7.**
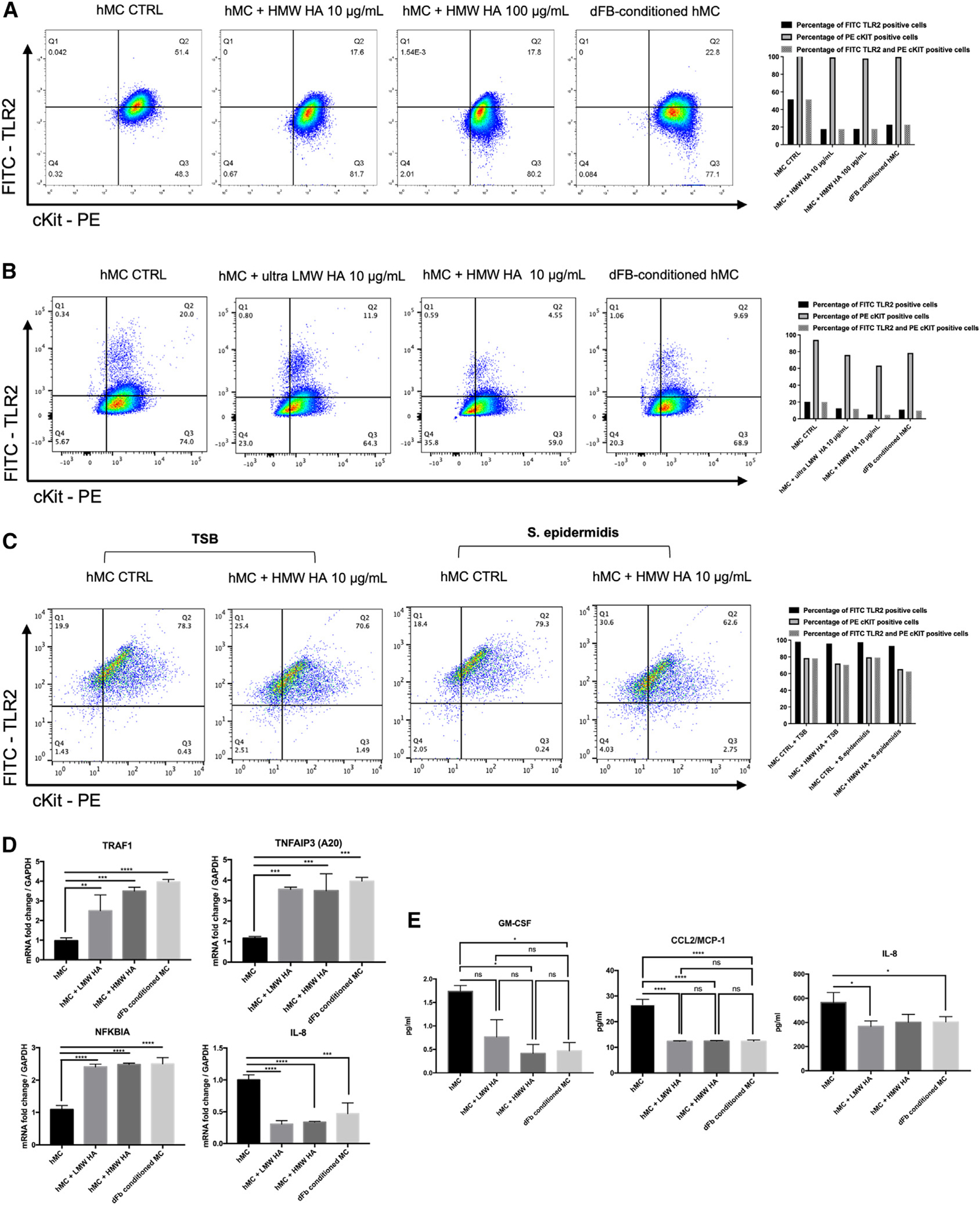
Hyaluronan (HA) inhibits MC cytokine release (A) Flow cytometry analysis of TLR2 expression of unconditioned HMCs, conditioned with high molecular weight HA (HMC+HMW HA 10 and 100 μg/mL) at two different concentrations or with dFBs (HMC-dFB). (B) Flow cytometry analysis of TLR2 expression of HMCs conditioned with ultra-low molecular weight HA (HMC+ultra-LMW HA10 μg/mL), high molecular weight HA (HMC+HMW HA 10 μg/mL), or dFBs (dFB-conditioned HMCs). (C) Flow cytometry analysis of TLR2 expression of HMCs unconditioned and treated with *S*. *epi* (HMC+ *S. epi*) or conditioned with HMW HA and treated with *S*. *epi* (HMC+HMW+*S. epi*). (D) qPCR analysis of the expression of TRAF1, TNFAIP3, NFKBIA, and IL-8 in unconditioned HMCs or HMCs conditioned with LMW and HMW HA (HMC+HMW HA) or dFBs (dFB-conditioned MCs). Statistical significance was calculated via unpaired ANOVA. *p < 0.05; **p < 0.01; ***p < 0.001 (n = 3). (E) Protein expression (ELISA) of CCL2, GM-CSF, and IL-8 in unconditioned HMCs (HMCs) or HMCs conditioned with HMW (HMC+HMW HA) and LMW HA (HMC+LMW HA) or dFBs (dFB-conditioned MCs). Statistical significance was calculated via unpaired ANOVA. *p < 0.05; **p<0.01; ***p < 0.001 (n = 3, triplicates).

Our data suggested that a component of the ECM mediates the changes we observed in dFB-conditioned MCs. Since CD44^32^ is the receptor for hyaluronic acids (HA), we hypothesized that the observed changes were due to HA interacting with MCs. Indeed, the addition of HA (10–100 μg) reduced TLR2 expression in MCs up to 60%, comparable to the decline we observed with the HMC-dFB co-culture (Figure 7A).

To investigate the effect of HA on the TLR2 pathway on dermal MCs, we co-cultured mature MCs with 10 μg/mL ultra-low molecular weight (LMW) HA, 10 μg/mL high molecular weight (HMW) HA, or dFBs for 5 days (Figure 7B). We tested different weights of HA because biological functions differ depending on the size of the HA molecule. HMW HA (>500 kDa) is anti-angiogenic, anti-inflammatory, and immunosuppressive, whereas LMW HA (10–500 kDa) is pro-angiogenic and pro-inflammatory.^33^ We found that the addition of LMW HA to conditioned MCs decreased CD44 expression even more than conditioning alone but that addition of HMW HA produced no significant change compared with HMC control (Figure S6). Assessment of the HA content of HMCs and conditioned HMCs and for HMW HA treatments on MCs and conditioned MCs revealed that HMCs minimally secrete HA, dFBs highly express HA, and co-cultures of HMCs and dFBs have a similar HA content as do dFBs, thus supporting our hypothesis that HA is secreted by dFBs and not HMCs in the co-culture system (Figure S7).

Consistent with that idea, MCs stained with TLR2 and c-Kit antibodies before flow cytometry showed that HMW HA reduced TLR2 expression on MCs to the same extent as dFB co-culture, whereas LMW HA had only a minor effect on TLR2 expression (Figure 7B).

To verify that a similar result was obtained when MCs were treated with commensal and pathogens, as noted in co-culture with dFB (Figure 1), we treated or pretreated HMCs with *S*. *epi* supernatant, each with or without preconditioning with HA, and assessed TLR2 expression. The bacterial supernatant greatly enhanced TLR2 expression, but preconditioning with HA strongly inhibited TLR2 expression, even in the presence of *S. epi* supernatant, thus mirroring the outcome for the expression of cytokines (Figure 7C).

To prove that HA was responsible for increasing the TNFAIP3 (A20) pathway, we used the same HA concentration that modulated TLR2 expression in dFB-conditioned HMCs and assessed the gene expression of TNFAIP3, TNFKBIA and TRAF1 and of IL-8 protein. We found increased expression for TNFAIP3(A20), TNFKBIA, and TRAF1 but decreased IL-8 expression in HMCs (Figure 7D), indicating activation of the anti-inflammatory A20 pathway.^34^ Moreover, MCP-1, GM-CSF, and IL-8 protein levels decreased in HMCs after treatment with HA (Figure 7E). These findings suggest that HA, via theTNFAIP3 (A20) pathway, inhibits inflammatory cytokine release by MCs.

## DISCUSSION

In this study, we obtained multiple novel findings: (1) MCs become tolerant to commensal bacteria but only when co-cultured with dFB. (2) dFBs generate HA within the ECM, which acts by inducing A20/TNFAIP3 expression and inhibits NF-κB and the release of inflammatory cytokines. (3) Crosstalk between MCs and dFBs profoundly alters the phenotypes of both cell types. (4) *Ex-vivo* experiment with human skin and *in vivo* mouse skin reconstitution with HMCs proves that HMC conditioning occurs *in vivo* in the skin. Thus, HA in the ECM appears to link MCs, dFBs, and commensal bacteria. These findings have important implications for dermal MC-driven inflammation and reveal a previously unrecognized mechanism that regulates MCs in the skin. The skin at large, specifically human skin, has many mechanisms to control MCs since their uncontrolled reactivity leads to MC activation and triggers inflammation and allergic symptoms.^35^

Multiple mechanisms have been identified that blunt MC activation in the dermis. It was recently reported that loss of the normal epidermal innervation or ablation of MrgprD-expressing neurons in mouse skin increases the expression of the MC activating receptor Mrgprb2, resulting in increased MC degranulation and cutaneous inflammation in disease models.^36^ Our results here are in line with such prior evidence showing that regulation of MC activity is key for maintaining cutaneous immune homeostasis.

The current findings show that dFB-conditioned HMCs (1) reduce the production of GM-SCF and MCP-1 proteins, which are essential recruiters for antigen-presenting cells, and (2) down-regulate pro-inflammatory cytokines (IL-6, IL-8, and Th2 cytokines IL-4, IL-5, IL-10, and IL-13). Together, these results imply that HMCs in the skin have a tolerant phenotype to prevent unnecessary inflammation when encountering commensal bacteria.

The interaction between MCs and skin microbiome has been shown to be essential: MCs in the absence of a microbiome do not mature adequately,^14^ and without the microbiome, the epidermal layer does not express and release stem cell factor (SCF), which is essential for MC maturation.^14^ Thus, MCs need the microbiome to mature correctly; in turn, the microbiome needs dFB to regulate MCs.

This study reveals an essential immune role for dFBs in the skin. This role has dramatically changed the prior view of fibroblasts as merely a passive mechanical part of the skin.^37^ dFBs are the factory for the ECM, in which glycosaminoglycans are a primary constituent.^38^ The skin ECM’s function has expanded from structural support to a more porous framework that adjusts to maintain homeostasis. The ECM provides ligands for cell surface receptors such as integrins, glycans, and TLRs and regulates cellular signaling and immune cell interactions.^38^ Our data show that dFBs influence MC phenotype in the dermal environment by downregulating TLR2 via HA.

We used papillary and reticular dFB mixed cell cultures (Figure S10). dFB expressed CD36^+^; CD39^+^ at the beginning of the culture, but these were largely lost during the expansion, resulting in a reticular dermal dFB phenotype (Figure S10). Despite the loss of papillary fibroblasts, the dFBs induced a tolerant phenotype in MCs. This reduced reactivity also applies to the supernatant of the pathogen *S*. *aureus.* When HMCs are stimulated with *S*. *aureus,* IL-17 is increased and not inhibited by dFB preconditioning, implying that HMCs can distinguish “friend from foe” (Figure S1). This ability may be due to *S*. *aureus* toxins that can directly activate MCs.^39^

To better understand the reciprocal modifications induced by MCs and dFB in co-culture, we analyzed their interaction by scRNA-seq. This analysis showed that the two cell populations modify each other, especially dFBs, whose phenotype switches by encountering MCs. MCs tend to inhibit gene expression. These “lost genes” are immune activation related, likely contributing to the decreased reactivity to bacteria.

Bulk RNA-seq identified genes modified in MCs by dFBs and focused on innate immunity-related genes that a microarray from FANTOM 5 data had shown to be essential in the skin MCs.^40^ We found that MCs conditioned with dFBs increase mediators that inhibit NF-κB activation, e.g., A20 and NFKBIA.

A main observation from the FANTOM 5 database analysis is that highly expressed genes (e.g., TNFAIP3 [A20], a known negative MC cytokine pathway) have decreased expression when MCs are expanded *in vitro.* By contrast, TNFAIP3 (A20) expression is increased in dFB-conditioned HMCs compared with unconditioned HMCs. The loss of TNFAIP3 (A20) affects HMC reactivity to bacteria and leads to enhanced inflammatory responses, while TNFAIP3 (A20) is a feedback inhibitor in dFB-conditioned HMCs and limits the secretion of inflammatory cytokines. The data thus reveal that TNFAIP3 (A20) plays a key role in the dFB-conditioned HMC mode and is also associated with a decrease in GM-CSF and MCP-1 expression.

TNFAIP3 (A20) is a potent anti-inflammatory protein inhibiting NF-κB signaling. Human mutations and mouse knockouts of the TNFAIP3/A20 gene implicate its role in inflammatory and autoimmune pathologies^28,29,41^ but detailed mechanistic understanding of A20 activation has been lacking. The current findings imply a previously unrecognized mechanism by which it controls inflammatory signaling in dermal MCs and its activation in MC by dFB-released HA. Our results have potential implications for dermal innate immunity. MC tolerance to Gram-positive commensal bacteria benefits the skin environment and promotes the maturation of MCs^14^ as well as maintaining regulatory T cells (Tregs).^29,42^ We found that A20 inhibits NF-κB-dependent production of IL-8 and other pro-inflammatory cytokines by interfering with a TLR-mediated signaling pathway, as previously observed in the airway epithelium.^43^

RNA-seq analysis of MCs conditioned with dFB and treated with bacterial supernatant, together with the subsequent analysis by Pathway.commons, shows a strong signature for pathways related to the ECM and the receptor CD44. Our *String* analysis indicates that CD44 connects ECM with TLR2. It has been reported that CD44 associates with TLR2 when stimulated by the TLR2 ligand zymosan and that the cytoplasmic domain of CD44 is crucial for its regulatory effect on TLR signaling.^44^ A similar mechanism may be operative in HMCs exposed to HA. The idea that HA is a ligand of TLR2 has been proposed and appears to be linked to tissue specificity.^45–47^ In our system, TLR2 disappears from the surface in the presence of HA. In parallel, TLR4 and CD44 receptors remain expressed, but co-culture with dFB decreased CD44. In the lung,^48^ LMW-HA fragments stimulate macrophage chemokine production through TLR4, an activation mediated by MyD88, which is not activated in our system. In joints, HA suppresses IL–1β-mediated NF-κB activation and expression of MMPs in a CD44-dependent manner as it binds to CD44 receptor.^32,48,49^

Mouse skin MCs do not express TLR2, which is correlated with low levels of retinoic acid in the mouse dermis.^16^ The addition of retinol in cultures allows mouse MCs to express TLR2, but when deprived or in co-culture with fibroblasts, its expression is reduced.^50,51^ A similar finding does not appear to apply to HMCs. Our system did not contain retinoic acid or serum in our cultures/co-cultures.

We found that stimulation of HMCs with HA altered MC receptors and the cytokine profile similarly to what we observed in dFB-conditioned HMCs. HA is known to enhance T cell migration.^52,53^ Therefore, dFBs produce an environment that influences Treg migration and proliferation and ensures immuno-tolerance.^53,54^ We identified a similar function of dFBs on HMCs.

In our studies in HMCs, HA blocks TLR2 signaling, while TLR4/LPS response is retained. Previous studies reported that LMW HA and highly polymerized HA elicited pro- and anti-inflammatory responses by modulating TLR4 and TLR2,^55^ observations partially akin to our findings. LMW HAs did not change TLR2 expression as well as HMW HAs, which also affected the NF-κB pathway more than the LMW HAs. HMCs treated with HAs did not internalize TLR4, leaving it available for activation, a finding that differs from what is observed in macrophages.^56^ A protective effect by HA was not seen in *CD44*^−/−^ or *TLR4*^*−*^*/*^*−*^ macrophages.^56^

In conclusion, this study demonstrates that co-culture of hMCs with dFBs induces changes to the hMC phenotype akin to what one observes *in vivo* in the dermis. These changes include TLR expression and reactivity to the microbiome. The data show that dFBs promote MC tolerance to commensal bacteria while maintaining response to more pathogenic bacteria. We found that HA produced by dFBs is responsible for these immunoregulatory effects, downregulating the expression of TLR2 and increasing TNFAIP3/A20. The increase of A20 blocks NF-κB in HMCs. HMCs are conditioned by dFBs also in other aspects, and in turn, dFBs are conditioned by HMCs. The net result of this dFB-HMC interplay is a decrease in release of pro-inflammatory cytokines. Thus, interactions of MCs with dFBs by HA can modulate inflammatory response of MCs by altering the expression of specific genes. This mechanism may be important in specific settings, such as aging, when the content of HA in the skin decreases^57^ and in conditions in which the skin is more sensitive and subject to irritation and itch. The current findings also suggest that HA stimulation may be a means to suppress MC responses in diseases that involve allergic reactions and wound formation.^58^

### Limitations of the study

This study describes a pathway by which a member of the ECM, HA, inhibits MC response to commensal bacteria and attenuates the response to skin pathogens via NF-κB pathway downregulation. We found that TLR2 suppression by HA leads to activation of TNFAIP3 (A20). As a consequence, commensals are better tolerated by HMCs. However, supernatant from *S*. *aureus* (an opportunistic/pathogen) increased IL-17 release, demonstrating that MCs can use different mechanisms to recognize pathogens that are TLR2 independent.^39^ Although we obtained evidence for this mechanism in *in vitro* studies with supernatants and in *ex vivo* studies with human cells, we could not perform experiments with humans to confirm this idea *in vivo.* Although this is a limitation, unless the skin is breached, MCs will mostly be in contact with commensal bacteria by-products, thus making our approaches representative of encounters in the skin. In addition, the skin reconstitution studies showed that our findings apply to normal skin. Even though we identified a new pathway for TLR2 downregulation, additional studies are required to establish if and how TLR2 downregulation, activation of the NF-κB inhibitory pathway, and HA and CD44 are interrelated.

Our focus here has been on the interaction between MCs and dFBs. We cannot exclude the influence from other cell types, such as neurons, which have been reported to downregulate MC activation during contact sensitization.^36^ Further studies will be needed to assess the interaction of neurons, dFBs, and MCs.

## STAR★METHODS

### RESOURCE AVAILABILITY

#### Lead contact

Further information and requests for resources and reagents should be directed to and will be fulfilled by the lead contact, Dr. Anna Di Nardo (adinardo@ucsd.edu).

#### Materials availability

This study did not generate new unique reagents.

#### Data and code availability

- Data availability

Single-cell RNA-seq and RNASeq data have been deposited at GEO as series GSE223177, GSE223178, GSE223179, and GSE223180 and are publicly available as of the date of publication. Accession numbers are listed in the key resources table.

- Code availability

This paper does not report original code.

- Any additional information required to reanalyze the data reported in this paper is available from the lead contact upon request.

### EXPERIMENTAL MODEL AND SUBJECT DETAILS Animal model

- NOD.Cg-*Prkdc*^*scid*^ *Il2rg*^*tm1Wji*^*/SzJ (NSG) mice (from Jackson lab) - all females over 12 weeks.*
- B6.Cg-Kit^*W-sh*^/HNihrJaeBsmJ *(Kit*^*W-sh*^*)* mice *(from Jackson lab) -* all females over 12 weeks.
- All animal protocols were reviewed and approved by the University of California San Diego (approval number: S10288)

#### Human model

- Ex-vivo live human skin (from Genoskin)
- Human cord blood *(from UCSD) -* de-identified healthy blood donors after obtaining written, informed consent drawn in accordance with the written approval of the Institutional Ethics Committee of the University of California San Diego (IRB: Project #190445X Use of human cord blood- derived mast cells to study innate immunity, UCSD human research protection program.)

#### Microbe (bacteria) strain

- S. epidermidis (from Gallo Lab)
- S. Aureus 113 (From Gallo Lab)

#### Primary cell cultures

- Primary Dermal fibroblasts (dFBs) (From American Type culture collection-ATCC)
- Human cord blood CD34^+^-derived mast cells *(human cord blood from UCSD)*
- Normal CD34^+^ Cells (from Astarte)

### METHOD DETAILS

#### Human cells

Human MCs (HMCs) were derived from human cord blood CD34+ cells (IRB: Project #190445X Use of human cord blood-derived mast cells to study innate immunity. From the University of California San Diego human research protection program) according to the protocol by Kirshenbaum and Metcalfe.^59^ Briefly, CD34+ cells were cultured in serum-free culture media (Stemline II, Sigma) containing antibiotic-antimycotic (Thermo Fisher), recombinant human stem cell factor (100 ng/ml, R&D Systems), recombinant human IL-6 (100 ng/ml, R&D Systems), and recombinant human IL-3 (20 ng/ml, R&D Systems, first week only). After 10 weeks, mature HMCs were consistently generated as confirmed by the expression of CD117 and FcεRI. Cell maturation was confirmed by metachromatic staining with toluidine blue and FACS analysis for ckit marker. The purity of HMCs was greater than 98%.

Primary Dermal fibroblasts (dFBs) were purchased from the American Type culture collection (ATCC). DFB were expanded and cultured in RPMI 1640 medium (Invitrogen) supplemented with 10% fetal bovine serum (Hyclone), 25 mM HEPES (pH 7.4), 4 mM L-glutamine, 0.1 mM, nonessential amino acids, 1 mM sodium pyruvate, antibiotic-antimycotic (Thermo Fisher). Cells were used from P1 to P4. The cell phenotype quantified by FACS is in Figure S4 during expansion.

HMCs and dFB co cultures were performed in a multiwell plate with a 0.3 μm filter and the dFBs in the upper chamber. The co-cultures were maintained for 7 days in the HMC medium. dFBs were removed from the HMCs culture before adding the different treatments and the media was changed before the treatments with bacterial supernatants.

HA at different concentrations and at low and high molecular weights was purchased from Fisher Scientific and added to the cell culture every day for 7 days.

#### Bacterial supernatant stock preparation

*S*. *epidermidis* (ATCC^®^ Catalog No.12228) and *S*. *Aureus* 113 (ATCC^®^ Catalog No. 35556^™^) were acquired from ATCC. *S*. *Epidermidis* 1457 was a kind donation from the Gallo Lab (UCSD). Preparation of bacterial cultures was performed as follows: Bacterial stocks frozen at − 80 °C in TSB (Sigma-Aldrich, St. Louis, MO) with 20% glycerol were inoculated into 5 mL TSB. The culture was aerated by shaking at 120 rpm at 37 °C and grown overnight.

To define a concentration of 10^6^, the bacteria grown overnight were quantified by a spectrophotometer to Measure OD600 by using UNICO 1100 (OD600 = 1 is equal to 3 × 10^8^ CFU/mL). The bacterial culture was then centrifuged at 4000rpm, 15min. and filtered sterile using 0.22 μm syringe filters (Fisher Scientific, Waltham, MA). Aliquots of the same supernatant were kept at − 80 °C.

#### Bacterial supernatant cell treatments

200 μL of bacterial supernatants were added to cells with a total volume of 500 μL medium with 10^5^ MCs on day one. A second dose of 200μL of supernatant was added on day 2. The cells were harvested on day 3 for qPCR and RNA-seq. Cell medium was collected for Luminex ELISA.

#### Mice

NOD.Cg*-Prkdc*^*scid*^ *Il2rg*^*tm1Wjl*^/SzJ (NSG) and B6.Cg-Kit^*W-sh*^/HNihrJaeBsmJ *(Kit*^*W-sh*^*)* mice were provided by Jackson Laboratory. NSG mice were cross-bred with *Kit*^*W-sh*^ mice, resulting in an immunodeficient and MC-deficient mouse strain for HMC reconstitution. All animal protocols were approved by the University of California San Diego (IACUC approval number: S10288). All mice were females over 12 weeks.

#### RNA sequencing

Purified RNA samples were submitted to the UCSD Institute for Genomic Medicine core facility for library preparation and high-throughput next-generation sequencing, according to a previous report.^60^ Total mRNA from HMCs and dFBs were prepared using the QIAGEN RNeasy Kit (Hilden, Germany). library prep. The mRNA libraries were generated using stranded mRNA with sequencing on the Illumina Hiseq4000, 75 bp single reads with all samples multiplexed on 1 lane of the Hiseq. to obtain 40–50 million reads per sample. Reads were mapped to the Hg38 human genome with STAR aligner.^61^ Subsequent analysis was conducted by using Partek^®^ installed on a Linux server at the Department of Dermatology at UCSD. The reference genome for alignment was Hg38 release 105 using STAR. Counts were normalized and analyzed with GSA. Parallel hierarchical clusters and heat maps were generated according to the feature lists. Parallel data were also analyzed using R to confirm the results. For R analysis, alignment-free quantification was performed via Kallisto of transcripts. Transcript expression converted via tximport in R. Differential gene expression (DE) in R was calculated via Sleuth or EDGER.

#### Single cell RNA sequencing

Following dilution in cold PBS, pelleting at 500×g for 8 min, and resuspension in cold 0.04% BSA/PBS, MCs and dFBs were counted. Viability was determined using a C-chip hemocytometer and trypan blue. Up to 10 million cells were used for MACS dead cell removal (Miltenyi; protocols). The flow through (live cells) was pelleted, resuspended in cold 0.04% BSA/PBS, and cell number/viability was determined using trypan blue and C-chip. Cells were loaded onto the 10× Genomics Chromium Controller following the single-cell 3 v2 protocol, aiming for between 2000 and 5000 cell recoveries. cDNA libraries were prepared from single-cell suspensions following the 10× Genomics 3 v2 protocol; 2 samples per lane were sequenced on HiSeq4000 with 26 bp read 1, 8 bp sample index, and 98 bp read 2 (aiming for 150 M reads/sample or ≥ 30,000 per cell). Reads were mapped to GRCh38 1.2.0 Human Genome reference by Cell Ranger 2.0.2 pipeline.

The workflow for single cell analysis included differential expression (DE) of genes expressed by MCs and dFBs in co-culture. Files were read from Cell Ranger at the core facility and channeled through R and Seurat; the readings were merged. Filter was applied for cells with too few or too many reads or too much mitochondrial stress-related gene expression. We also filtered out genes detected in less than 100 cells. We Identified genes/markers which appeared to account for high heterogeneity, i.e., which denote cell types. Finally, we renormalized data, accounting for factors such as library size using the SC-transform tool. Visualization was done with UMAP to identify clusters. We used Cell Selector with the UMAP cluster plots to mark cells as a particular type for downstream analysis. We imported the resulting transformed counts’ files, to edgeR^62^ for DE analysis. For DE genes, pathway analysis was performed using Enrichr.^63^ Basic statistics are described in Figure S2.

Using Fisher’s exact test, we compared the detected and undetected contingency tables for both groups. This non-parametric test is most appropriate, as it carries the fewest assumptions about the underlying statistical behavior of the data, assumptions that make it inappropriate to use certain other statistical analyses, such as T-tests. The approach we used yields odds ratios that indicate whether a gene is more likely to be found in one group or the other and gives a p-value for each gene. Values were adjusted for multiple testing in R programming, using False discovery rates (FDRs); FDRs < 0.01 were considered significant. Therefore, we tested for the presence/absence of genes between groups, rather than differences in quantity, due to the largely binary nature of the output data The details are in Figure S2.

#### Real-time quantitative RT-qPCR

Total RNA of cells was isolated by Rneasy Mini Kit (QIAGEN). At least 1 μg of total RNA was used for cDNA synthesis by the iSCRIPT cDNA Synthesis Kit (Bio-Rad) according to the manufacturer’s instructions. Real-time quantitative polymerase chain reaction (RT-qPCR) was performed in an BioRad real-time PCR system. All primers and probes used for RT-qPCR were purchased from Applied Biosystems. RNA RT-PCR samples were prepared by using the TaqMan Master Mix reagents kit (Applied Biosystems). Gene expression analysis were calculated with ΔΔCT method to determine the quantification of gene expression. We normalized the target gene expression in the test samples to levels of the endogenous reference, Glyceraldehyde 3-phosphate dehydrogenase (GAPDH), and reported them as the fold-difference relative to GAPDH gene expression in untreated baseline controls.^64^ All assays were performed with at least triplicate samples, and experiments were repeated more than two times.

#### Fluorescence-activated cell sorting (FACS)

Single-cell suspensions were prepared by mincing mouse skin tissue with scissors, followed by enzymatic digestion with collagenase Type II (Worthington), collagenase Type IV (Gibco), and 0.53 mg/ml Dnase I (Roche). Cells were stained with anti-CD117, anti-FcεRI, or anti-TLR2 monoclonal antibodies (BD Biosciences) according to the manufacturer’s instructions. Cells were analyzed with the Guava EasyCyte 8HT two laser, 6 color microcapillary-based benchtop flow cytometer (Millipore). FlowJo v10 (Treestar, Ashland, OR) produced the flow cytometry plots.

#### Elisa and luminex assay

HMCs and HMC co-cultured with human dFBs were treated with PBS, 10 μL LTA or 2μL LPS and incubated overnight. Cell supernatants were collected to determine cytokine protein concentration using either Human Luminex Discovery Assay (R&D Biosystems) or DuoSet ELISA kit (R&D) according to the manufacturer’s instructions. Luminex assays were analyzed on a MAGPIX instrument (Luminex Corporation) and ELISA was analyzed on Spectra Max ID3.

#### Genoskin

*Ex-vivo* live human skin assays were purchased from Genoskin Inc. Subcutaneous injection of 1×10^6^ MCs was performed according to the manufacturer’s instructions. Then 100μL PBS, TSB or S*.epidermidis* were applied topically and incubated for 4 days, after which, skin biopsies were sent to UCSD Moores Cancer Center for paraffin embedding.

#### Immunofluorescence of skin and HMCs

Skin sections were immunostained, as described previously,^14^ with the primary and secondary antibodies listed in STAR Methods. Fluorescence images were obtained with a fluorescence microscope (Olympus BX51). HMCs were stained with FITC-conjogated anti TLR2 antibody (Invitrogen).

### QUANTIFICATION AND STATISTICAL ANALYSIS

In all *in vitro* experiments, all samples were performed in triplicates, and values are expressed as mean ± standard deviation. Mann-Whitney test were applied to analyze the differences between the two groups. One- or two-way analysis of variance (ANOVA) and Tukey tests were applied to analyze the differences among more than two groups with a confidence level of 95%. Data from RNA seq were analyzed in Partek^®^ using GSA (GSA stands for gene-specific analysis, the goal of which is to identify the statistical model that is the best for a specific gene among all the selected models, and then use that best model to calculate the p-value and fold change) and ANOVA.

A list of the reagents can be found in the supplement material.

## Supplementary Material

1

## Figures and Tables

**KEY RESOURCES TABLE:** 

REAGENT or RESOURCE	SOURCE	IDENTIFIER

Antibodies		

APC anti-mouse CD117 (c-kit) Recombinant Antibody	BioLegend	155107
APC anti-human FcεRIα Antibody	BioLegend	334612
BV711 Mouse Anti-Human CD117	BD Biosciences	740831
BB700 Mouse Anti-Human FcεR1α	BD Biosciences	747780
BUV395 Mouse Anti-Human CD282 (TLR2)	BD Biosciences	742772
Texas Red^™^ Avidin D	Vector Laboratories	A-2006-5
FITC anti-human CD44 Antibody	BioLegend	397517
PE anti-mouse CD117 (c-Kit) Antibody	BioLegend	105807
APC anti-human MRGX2 Antibody	BioLegend	359005
APC anti-human CD324 (E-Cadherin) Antibody	BioLegend	324107
FITC anti-human CD45	BioLegend	982316
APC anti-human CD31 Antibody	BioLegend	303115
FITC anti-human CD36 Antibody	BioLegend	336203
FITC anti-human CD39 Antibody	BioLegend	328205
APC anti-human CD90 (Thy1) Antibody	BioLegend	328113
FITC anti-human CD282 (TLR2) Antibody	BioLegend	392307
CD284 (TLR4) Monoclonal Antibody (HTA125), eBioscience	ThermoFisher	14-9917-82
TLR2 Recombinant Rabbit Monoclonal Antibody (JM22-41)	ThermoFisher	MA5-32787
PE anti-mouse CD282 (TLR2) Antibody	BioLegend	148603
Anti-Mast Cell Chymase antibody	Abcam	Ab233103
DAPI (4′,6-Diamidino-2-Phenylindole, Dilactate)	BioLegend	422801
Recombinant Anti-TLR2 antibody [EPR20302-119]	Abcam	Ab209216

Bacterial and virus strains		

*Staphylococcus epidermidis*(SE) ATCC12228	Gallo lab (UCSD)	N/A
*Staphylococcus aureus SA113 Rosenbach*	Gallo lab (UCSD)	N/A

Chemicals, peptides, and recombinant proteins		

Recombinant Human SCF Protein	R&D Systems	255-SC-200/CF
Recombinant Human IL-6 Protein	R&D Systems	206-IL-200/CF
Recombinant Human IL-3 Protein	R&D Systems	203-IL-100/CF
HyClone Characterized Fetal Bovine Serum (FBS), U.S. Origin	HyClone	SH30071.03
HEPES (1 M)	ThermoFisher	15630080
L-Glutamine (200 mM)	ThermoFisher	25030081
Sodium Pyruvate (100 mM)	ThermoFisher	11360070
RPMI 1640 Medium	Invitrogen	11-875-085
Antibiotic-Antimycotic (100X)	ThermoFisher	15240096
PBS, pH 7.4	ThermoFisher	10010023
Bovine Serum Albumin (BSA) Powder–Serum Replacement Grade	GeminiBio	SKU# 700-100P-100
Trypan Blue Solution, 0.4%	ThermoFisher	15250061
Dead Cell Removal Kit	Miltenyi	130-090-101
RNeasy Mini Kit (250)	Qiagen	74106
iScript^™^ cDNA Synthesis Kit, 100 × 20 μL rxns #1708891	Bio-Rad	1708890
Biorad real-time PCR system	Bio-Rad	N/A
Collagenase, Type II	Worthington	LS004176
Collagenase, Type IV, powder	Gibco	17104019
Dnase	Roche	SKU 4716728001
Lipopolysaccharide (LPS)	Invivogen	14D9
Human CD117/c-kit DuoSet ELISA	R&D Biosystems	DY332
Human CCL2/MCP-1 DuoSet ELISA	R&D Biosystems	DY279
Human GM-CSF DuoSet ELISA	R&D Biosystems	DY215
Human IL-8/CXCL8 DuoSet ELISA	R&D Biosystems	DY208
Toluidine blue	Sigma- Aldrich	89640-5G
Stemline^®^ II Hematopoietic Stem Cell Expansion Medium	Sigma-Aldrich	S0192
Hyaluronan (High MW) 100MG	Sigma-Aldrich	GLR002
Hyaluronan (Low MW) 100MG	Sigma-Aldrich	GLR001
Hyaluronan DuoSet ELISA	R&D Biosystems	DY3614-05
Lipoteichoic acid from S. aureus	InvivoGen	tlrl-slta

Deposited data		

RNA seq triplicate with commensal bacteria	GSE223180	NCBI NIH
RNA seq duplicate for LTA	GSE223180	NCBI NIH
Sc RNA seq Dermal fibroblasts and Mast cells	GSE223180	NCBI NIH

Experimental models: Cell lines		

Primary Dermal Fibroblast; Normal, Human, Adult (HDFa)	American Type culture collection (ATCC)	PCS-201-012
Human ex-vivo skin	GenoSkin	order.accessusa@genoskin.com
Normal CD34^+^ Cells	Astarte	1014-759AP11
CD34+ cells from human cord blood	UCSD	IRB: Project #190445X

Experimental models: Organisms/strains		

B6.Cg-KitW-sh/HNihrJaeBsmJ (KitW-sh)	Jackson Lab	Strain #012861; RRID:IMSR_JAX:012861
NOD.Cg-*Prkdc^scid^Il2rg^tm1Wjl^*/SzJ (NSG)	Jackson Lab	Strain #005557; RRID:IMSR_JAX:005557

Oligonucleotides		

TRAF1 TaqMan^™^ Gene Expression Assay (FAM)	Applied Biosystems	4453320
TNFAIP3	Applied Biosystems	4453320
NFKBIA	Applied Biosystems	4453320
IL-8 (CXCL8)	Applied Biosystems	4453320
TLR2	Applied Biosystems	4453320
TLR4	Applied Biosystems	4453320
GAPDH	Applied Biosystems	4453320

Software and algorithms		

Partek Flow Build version 10.0.22.0330	Partek	https://www.partek.com/
FlowJo v.10.5.3	TreeStar	FlowJo LLC Oregon
GraphPad Prism 9.4.1	GraphPad	GraphPad Prism 9.4.1 (458)
Pipeline Version: cellranger-6.0.0	Loupe cell browser	10X genomics
R analysis, alignment-free quantification was performed via Kallisto of transcripts. Transcript expression converted via tximport in R. Differential gene expression (DE) in R was calculated via Sleuth or EDGER.	N/A	R Statistical Software (v4.1.2; R Core Team 2021)

Other		

Millipore Guava EasyCyte 8HT Flow Cytometer	Millipore	12568
Fluorescence microscope	Zeiss	N/A
Reichert hemocytometer	Hausser scientific	02-671-5
Spectra Max ID3	Molecular devices	N/A
Luminex 200 RUO System w/xPONENT 4.3	Luminex	LX200-XPON-RUO
MAGPIX System	Luminex	MAGPIX System
FLEXMAP 3D with xPONENT 4.2	Luminex	FLEXMAP-3D-RUO

## Highlights

- Human mast cell (HMC) TLR2 expression is reduced into the skin
- scRNA-seq shows that HMC and dermal fibroblast gene expression are reciprocally influenced
- Dermal fibroblast upregulates A20, a NF-κB inhibitory gene that reduces HMC reactivity
- The extracellular matrix component, hyaluronan, decreases mast cell cytokine release

## INCLUSION AND DIVERSITY

We support inclusive, diverse, and equitable conduct of research.
