# Supplementary material for: Mast cell tolerance in the skin microenvironment to commensal bacteria is controlled by fibroblasts
Source: Cell Rep. Author manuscript; Available in PMC 2023 Jun 24. (PMC10290424; doi:10.1016/j.celrep.2023.112453)

Cell Reports, Volume *42*

Supplemental information

Mast cell tolerance in the skin microenvironment

to commensal bacteria is controlled by fibroblasts

Anna Di Nardo, Yu-Ling Chang, Shahrzad Alimohammadi, Kana Masuda-Kuroki, Zhenping Wang, Krishna Sriram, and Paul A. Insel

SUPPLEMENTARY FIGURE LEGENDS

Supplementary Figure S1. Cytokine expression modulation of HMC and conditioned HMC upon TLR2 and TLR4 ligands (upper panel) as well as the addition of bacteria (lower panel).

Based on ELISA results, cytokine levels of IL-1α, IL-1β, IL-1RA, IL-2, CXCL10, TNF-α, IL-17A, IL-12 (P40), and IL-12 (P70) were elevated upon LPS and LTA treatments. Significant changes in the protein expressions of the same proteins with the addition of S. Epidermidis and S. aureus were seen in the case of HMC and conditioned HMC. The data shown represent the Mean±SD (n=3). Statistical significance was calculated via unpaired ANOVA. *= P< 0.05 ** =P<0.01. *** =P<0.001.

Supplementary Figure S2. Statistics related to the single cells RNA seq and most significant gene changes in HMCs and dFB when cultured alone or in co-culture.

Both cell populations represented in UMAP1 and UMAP2 show a more pronounced shift in the dFB population. The most significant genes in either population changed by co- culture. The plots represent the number of genes / mRNAs detected per cell, the Number of counts per cell, i.e. the total number of detected RNA molecules per cell, and the % of counts that correspond to mitochondrial stress-associated genes, respectively.

Supplementary Figure S3. Volcano plots of up-regulated, down-regulated, and no significant difference of Control HMC, dFB conditioned HMC, HMC treated with bacterial supernatant, and dFB conditioned HMC treated with bacterial supernatant. X-axis presents fold change, and the Y-axis shows P-values. Partek® software was used for data analysis.

Supplementary Figure S4. Principal component representation for dFB conditioned by MCs, dFB alone, HMC alone and HMC conditioned with dFB

where X-axis represents PC1 and PC3, while Y-axis shows PC2. Partek software was used for data analysis.

Supplementary Figure S5. DE analysis of HMC increased and decreased genes by co- culture. HMCs show reduced expression of genes/pathways typically associated with immune activation and function of MCs and other immune cell types. Differential Expression analysis results for MCs showed that the number of significant genes (FDR < 0.01) was 8840. Significant and Up >2 fold due to co-culture =15; Significant and Down Up >2 fold due to co-culture = 25; Significant and Up >50% due to co-culture = 59; Significant and Down >50% due to co-culture = 2172. Enrichment sore for association with specific cell and tissue types.

Supplementary Figure S6. Flow cytometry analysis of HMC treated with HA. Measurements revealed that CD44 expression is decreased in the case of conditioned HMCs compared to HMCs alone. Adding LMW HA to conditioned mast cells decreased CD44 expression compared to conditioned alone, while adding HMW HA showed no significant change compared to HMCs control.

Supplementary Figure S7. Hyaluronan (HA) ELISA quantification of mast cells medium before and after dFB conditioning and HA treatment. As presented, HMCs minimally secrete HA while dFB highly expresses this protein, and the co-culture of HMCs and DFs represent similar HA content compared to dFBs. This confirms that HA is only secreted through dFBs, not HMCs in the co-culture system. The data shown represent the Mean±SD (n=3). Statistical significance was calculated via unpaired ANOVA. *= P< 0.05. ** =P<0.01. *** =P<0.001

Supplementary Figure S8. mRNA expressions of TLR2 and TLR4 in HMC and conditioned HMC. The data shown represent the Mean±SD (n=3).

Statistical significance was calculated via unpaired ANOVA. *= P< 0.05 ** =P<0.01.   *** =P<0.001.

Supplementary Figure S9. TLR2 expression in HMC in vitro upon TLR2 ligands stimulation, including LTA, SE12228, Pam2CSK4, and Zymosan. AF488 FITC presents TLR2, and blue DAPI represents the nucleus. Fluorescence is diffused in PBS treated and is para nuclear in the treated cells. Scale bar 50µm.

Supplementary Figure S10. FACS representation of the phenotype of the dFBs that were used to condition HMC by FACS analysis.

Supplemental Figure S1

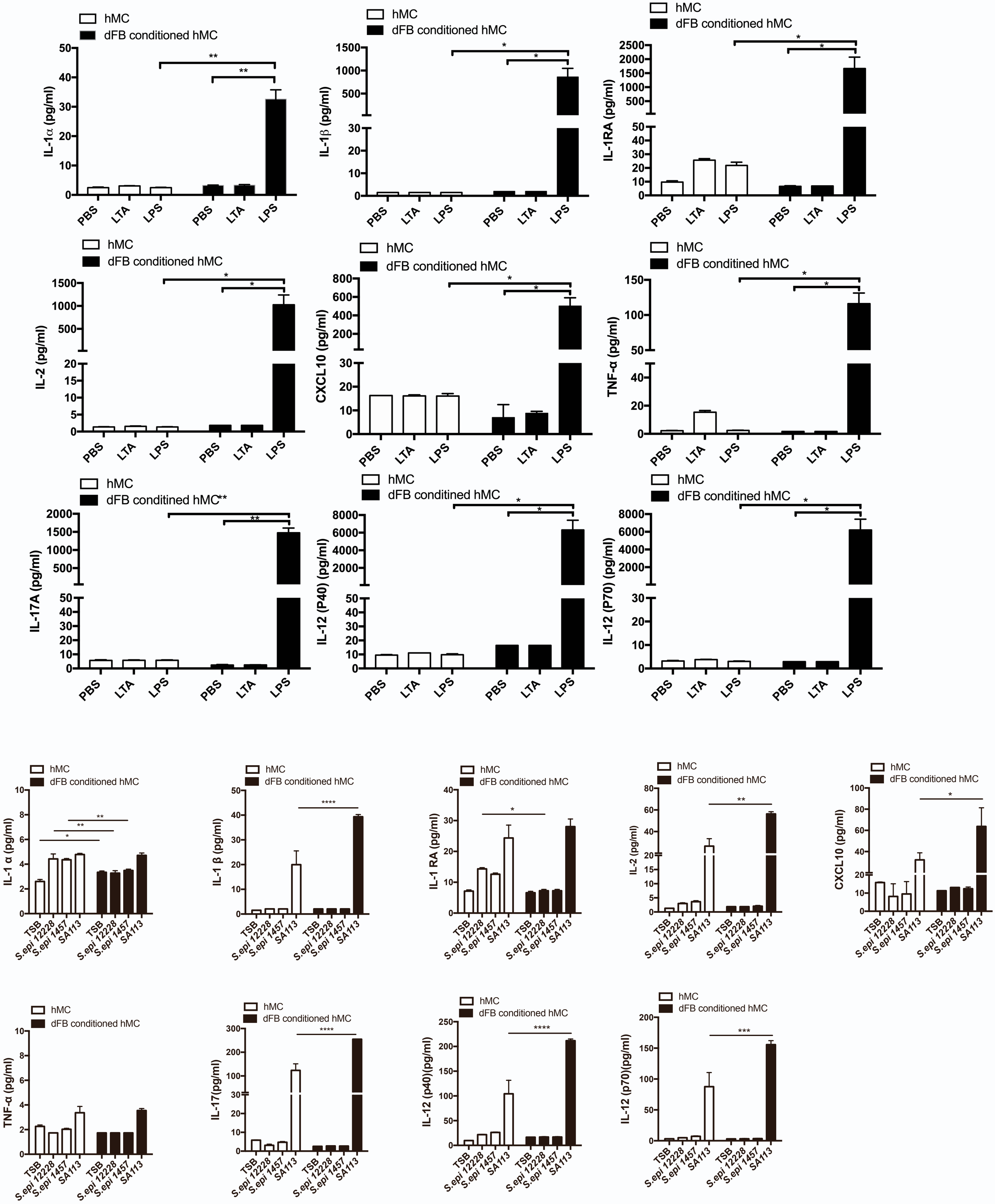

Supplemental Figure S2

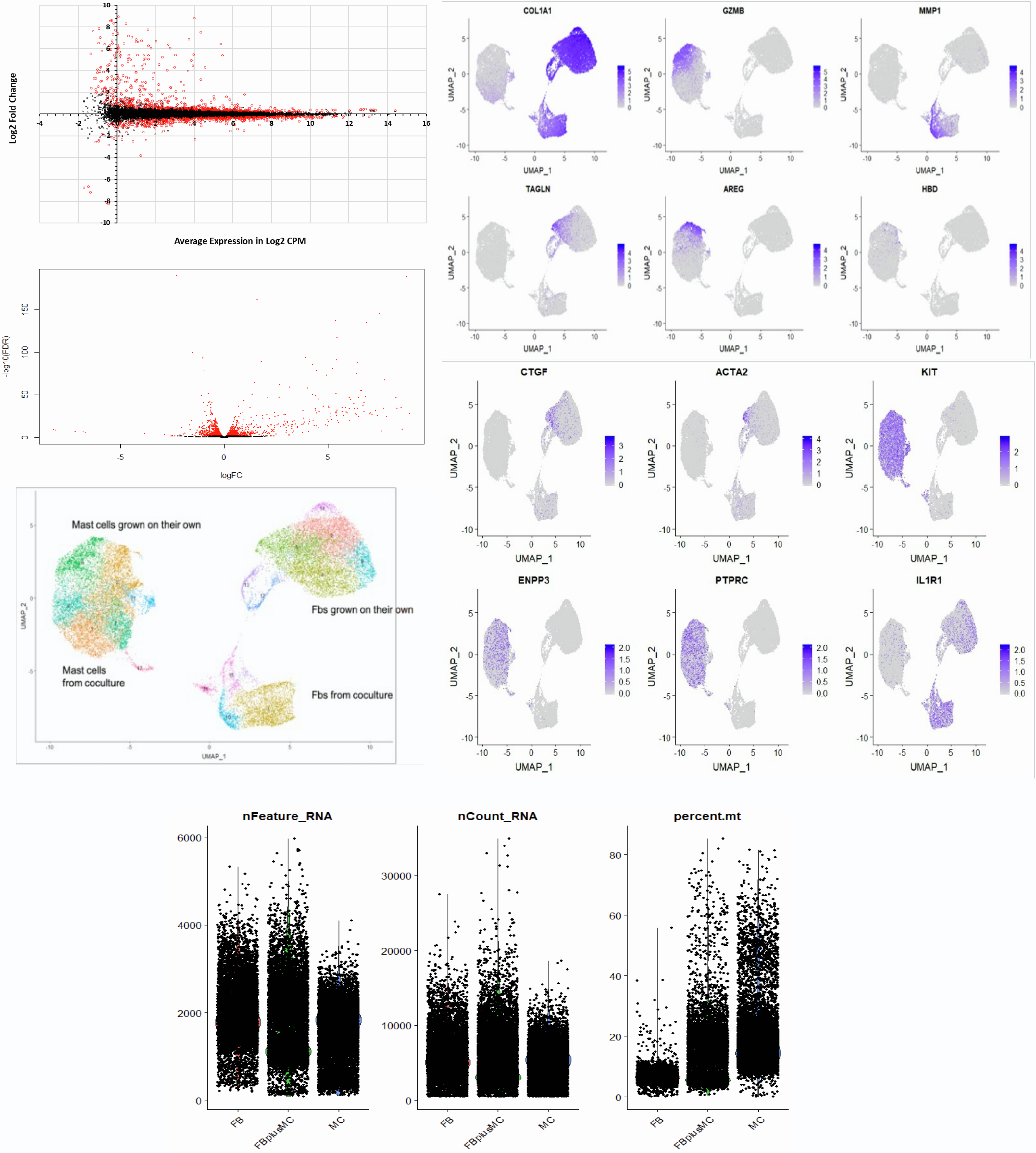

The number of genes / mRNAs detected per cell

Number of counts per cell, i.e. the total number of detected RNA molecules per cell

The % of counts that correspond to mitochondrial stress-associated genes, usually implies dead cells if high

Supplemental Figure S3

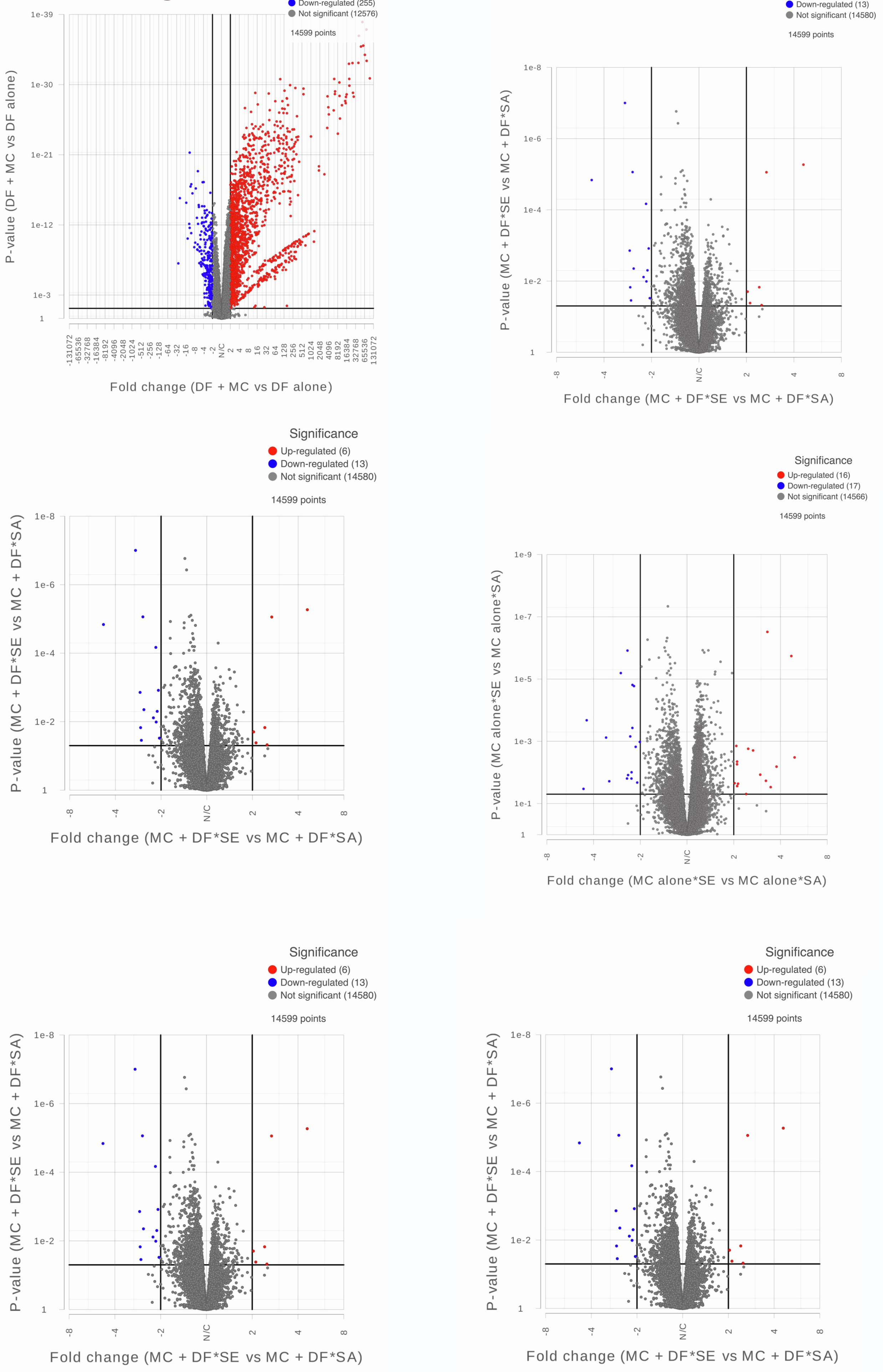

Supplemental Figure S4

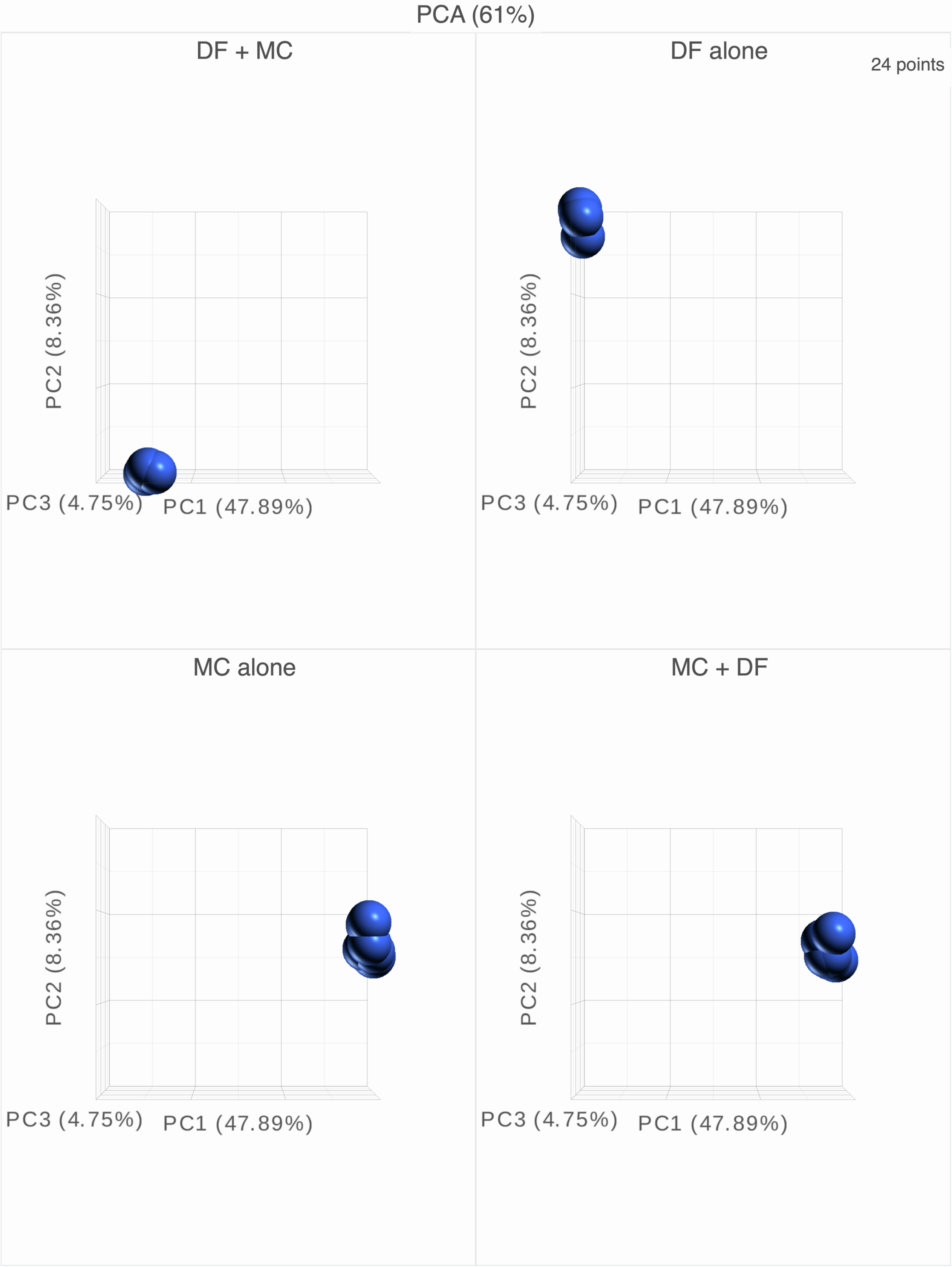

Supplemental Figure S5

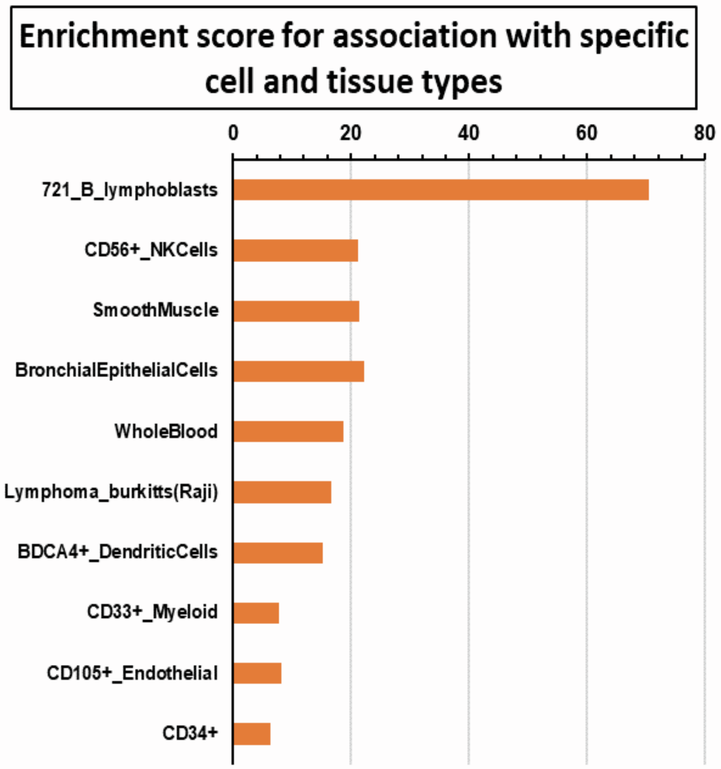

Supplemental Figure S6

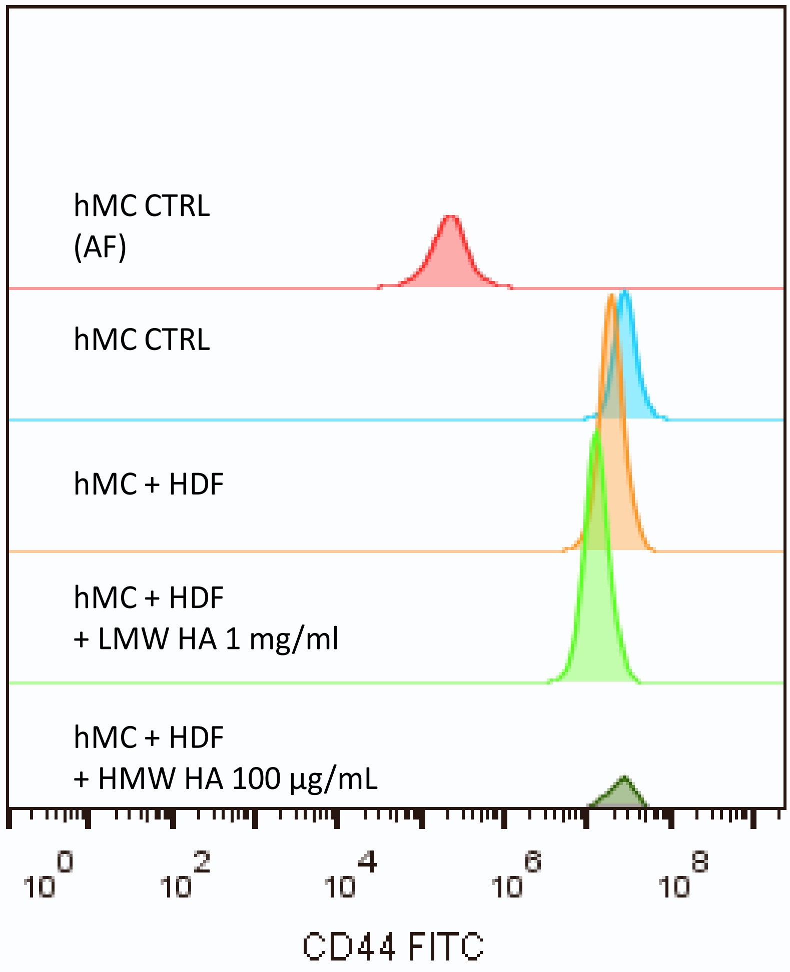

Supplemental Figure S7

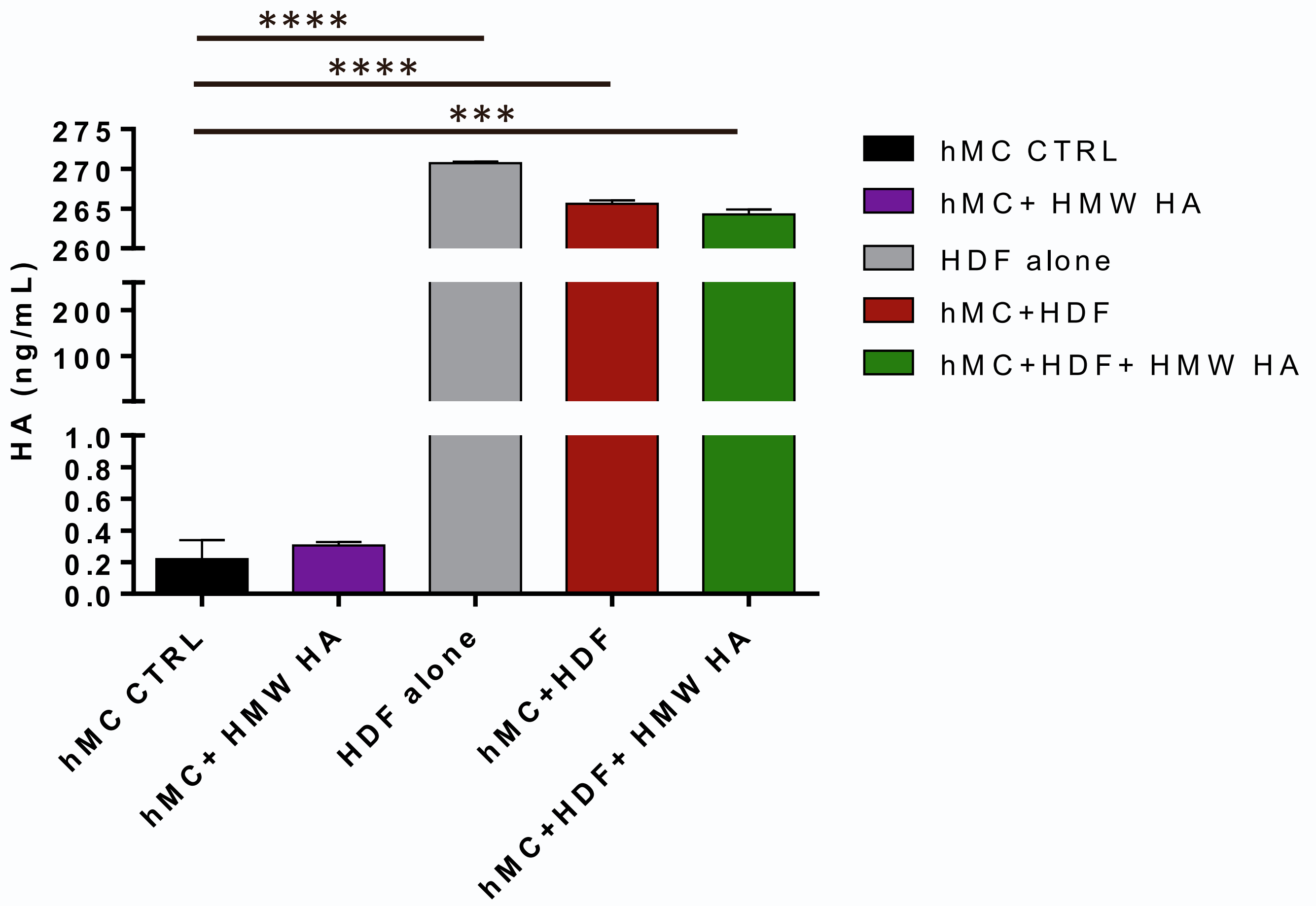

Supplemental Figure S8

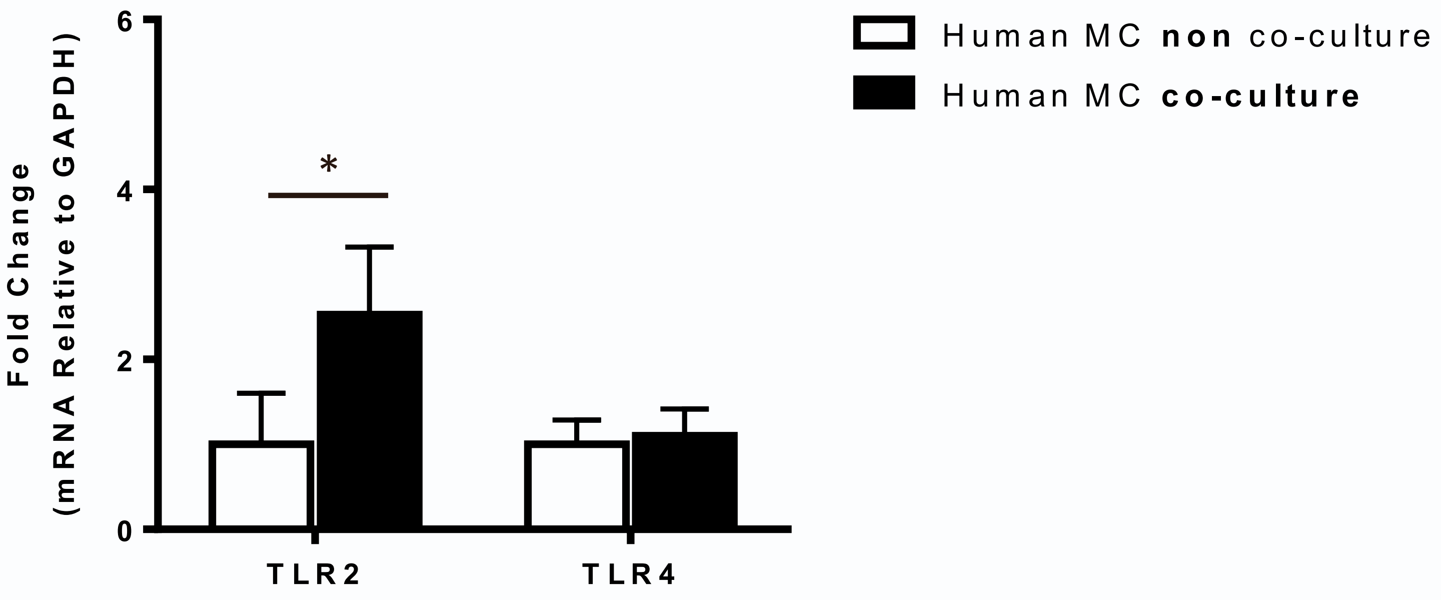

Supplemental Figure S9

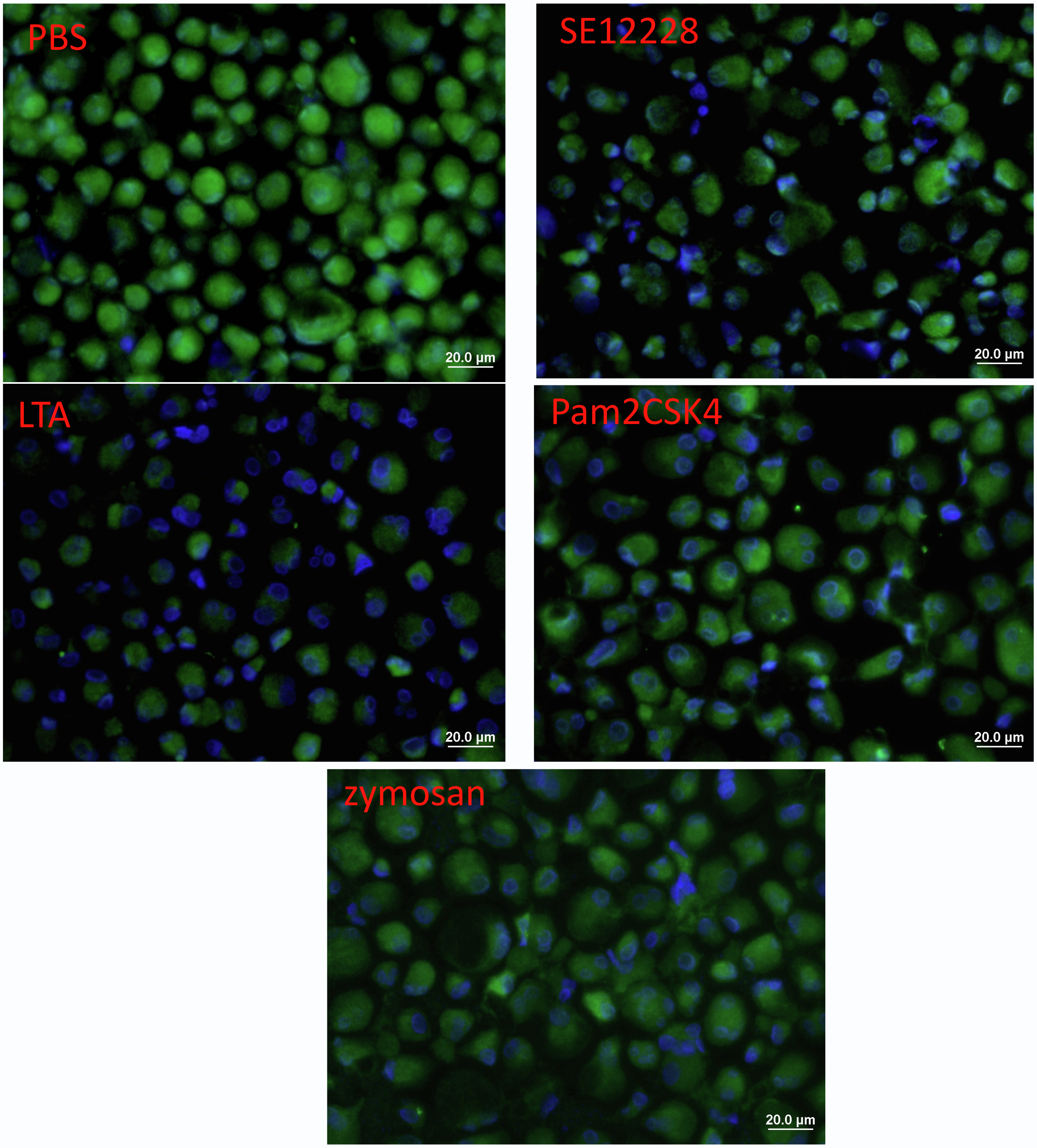

Supplemental Figure S10

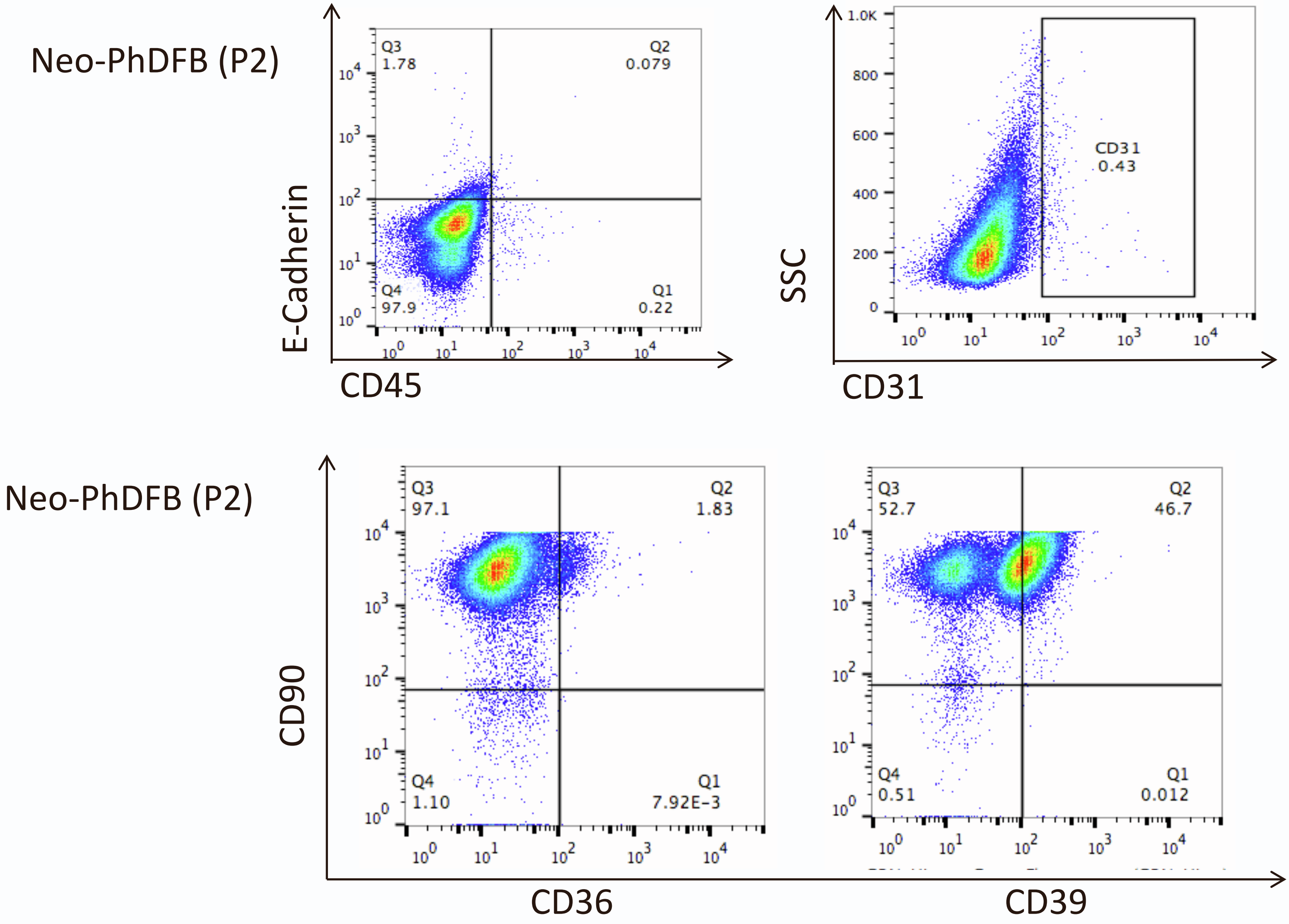

Supplement: 1 [file NIHMS1905366-supplement-1.pdf]
